# Beneficial Effect of Faecal Microbiota Transplantation on Mild, Moderate and Severe Dextran Sodium Sulphate-Induced Ulcerative Colitis in a Pseudo Germ-Free Animal Model

**DOI:** 10.3390/biomedicines12010043

**Published:** 2023-12-22

**Authors:** Stanislav Lauko, Sona Gancarcikova, Gabriela Hrckova, Vanda Hajduckova, Zuzana Andrejcakova, Livia Kolesar Fecskeova, Izabela Bertkova, Emilia Hijova, Anna Kamlarova, Martin Janicko, Lubos Ambro, Monika Kvakova, Zuzana Gulasova, Ladislav Strojny, Gabriela Strkolcova, Dagmar Mudronova, Marian Madar, Vlasta Demeckova, Daniela Nemetova, Ivan Pacuta, Drahomira Sopkova

**Affiliations:** 1 Department of Microbiology and Immunology, University of Veterinary Medicine and Pharmacy in Kosice, 041 81 Kosice, Slovakia; stanislav.lauko@uvlf.sk (S.L.); vanda.hajduckova@uvlf.sk (V.H.); dagmar.mudronova@uvlf.sk (D.M.); marian.madar@uvlf.sk (M.M.); daniela.nemetova@student.uvlf.sk (D.N.); ivan.pacuta@student.uvlf.sk (I.P.); 2 Institute of Parasitology, Slovak Academy of Sciences, 041 81 Kosice, Slovakia; hrcka@saske.sk; 3 Department of Biology and Physiology, University of Veterinary Medicine and Pharmacy in Kosice, 041 81 Kosice, Slovakia; zuzana.andrejcakova@uvlf.sk (Z.A.); drahomira.sopkova@uvlf.sk (D.S.); 4 Associated Tissue Bank, Faculty of Medicine, Pavol Jozef Safarik University and Louis Pasteur University Hospital (UHLP) in Kosice, 040 11 Kosice, Slovakia; livia.kolesar.fecskeova@upjs.sk; 5 Center of Clinical and Preclinical Research—MEDIPARK, Faculty of Medicine, Pavol Jozef Safarik University in Kosice, 040 11 Kosice, Slovakia; izabela.bertkova@upjs.sk (I.B.); emilia.hijova@upjs.sk (E.H.); anna.kamlarova@upjs.sk (A.K.); monika.kvakova@upjs.sk (M.K.); zuzana.gulasova@upjs.sk (Z.G.); ladislav.strojny@upjs.sk (L.S.); 6 2nd Department of Internal Medicine, Faculty of Medicine, Pavol Jozef Safarik University and Louis Pasteur University Hospital in Kosice, 040 11 Kosice, Slovakia; martin.janicko@upjs.sk; 7 Center for Interdisciplinary Biosciences, Technology and Innovation Park, Pavol Jozef Safarik University in Kosice, 040 01 Kosice, Slovakia; lubos.ambro@upjs.sk; 8 Department of Epizootiology, Parasitology and Protection of One Health, University of Veterinary Medicine and Pharmacy in Kosice, 041 81 Kosice, Slovakia; gabriela.strkolcova@uvlf.sk; 9 Department of Animal Physiology, Institute of Biology and Ecology, Faculty of Science, Pavol Jozef Safarik University in Kosice, 040 01 Kosice, Slovakia; vlasta.demeckova@upjs.sk

**Keywords:** faecal microbiota transplantation, pseudo germ-free mice, DSS, mild, moderate, severe ulcerative colitis

## Abstract

Transplantation of faecal microbiota (FMT) is generally considered a safe therapeutic procedure with few adverse effects. The main factors that limit the spread of the use of FMT therapy for idiopathic inflammatory bowel disease (IBD) are the necessity of minimising the risk of infection and transfer of another disease. Obtaining the animal model of UC (ulcerative colitis) by exposure to DSS (dextran sodium sulphate) depends on many factors that significantly affect the result. *Per os* intake of DSS with water is individual for each animal and results in the development of a range of various forms of induced UC. For this reason, the aim of our study was to evaluate the modulation and regenerative effects of FMT on the clinical and histopathological responses and the changes in the bowel microenvironment in pseudo germ-free (PGF) mice of the BALB/c line subjected to chemical induction of mild, moderate and serious forms of UC. The goal was to obtain new data related to the safety and effectiveness of FMT that can contribute to its improved and optimised use. The animals with mild and moderate forms of UC subjected to FMT treatment exhibited lower severity of the disease and markedly lower damage to the colon, including reduced clinical and histological disease index and decreased inflammatory response of colon mucosa. However, FMT treatment failed to achieve the expected therapeutic effect in animals with the serious form of UC activity. The results of our study indicated a potential safety risk involving development of bacteraemia and also translocation of non-pathogenic representatives of bowel microbiota associated with FMT treatment of animals with a diagnosed serious form of UC.

## 1. Introduction

Idiopathic inflammatory bowel disease (IBD) is a chronic recurrent inflammatory bowel disease with global occurrence, characterised by disturbances in the intestinal barrier and accompanied by changes in the microbial community, systemic biochemical abnormalities [1] and immune dysfunction [2,3]. Although the aetiology of this disease still remains unclear, it has been generally agreed that IBD involves interactions among the genetic makeup of the patient, environmental factors, gut microbiota and the immune system [4,5]. IBD occurs in two major clinical forms, ulcerative colitis (UC) and Crohn’s disease (CD), that differ by the clinical manifestations of inflammation and its localisation and are characterised by unique microbial signatures.

Dysfunction and disintegration of the intestinal barrier play a key role in the initiation and progression of intestinal inflammatory diseases. Damage to the epithelial barrier results in increased penetration and absorption of microbial and immunogenic factors. Stimulation of immunocompetent cells leads to the production of pro-inflammatory cytokines (TNF-α, IFN-ƴ, IL-1β), which contribute to ulceration and mucosal degradation [6]. The concept of changes in gut microbiota in IBD pathology has been generally accepted and confirmed by clinical and experimental analyses [7,8,9,10] that imply that the intestinal microbiome and dysbiosis also play a key role in pathogenesis and initiation of inflammatory bowel disease; however, it still remains unclear whether the dysbiotic microbiota of patients can be considered the cause or only a simple reflection of inflammatory and antimicrobial responses induced in the course of disease [11]. Conventional methods of IBD therapy include medicamentous therapy and surgical interventions aimed at diminishing inflammation; however, their low effectiveness and high risk of side effects inevitably calls for the development and implementation of new, highly effective alternative treatment strategies or a combination of medicines with less toxic side effects that could induce or maintain the remission. Manipulation of the microbiome constitutes a potential therapy for diseased individuals with a changed composition of gut microbiota [12,13]. The success of such an approach demonstrates the significant role of microbiota in numerous diseases [14,15,16]. Microbiota replacement therapy or faecal microbiota transplantation (FMT) is a non-immunosuppressive treatment that, in essence, means transplantation or transfer of populations of faecal microorganisms from healthy individuals to patients with the aim of eliminating dysbiosis and re-colonising the gastrointestinal tract of the recipient with quantitatively and qualitatively unimpaired microbiota [17]. The key advantage of FMT in comparison with other forms of therapeutic manipulation with gut microbiota (such as the use of antibiotics, probiotics or prebiotics) is that it provides a complete functional ecosystem comprising a total spectrum of microorganisms from a healthy individual and thus has the potential to correct the so far non-characterised dysbiosis and functional disorders critical for IBD pathogenesis [18]. The idea of using FMT as a full-value therapeutic approach is not a new one. The oldest written mention of the benefits of consumption of *faeces* as a medicine dates from the 8th century B.C.; it was found in an ancient tomb in central China [19] and is known mainly to cultures in Asia and the Near East [20]. However, FMT did not acquire the status of a recognised therapeutic procedure until 2013, when the Food and Drug Administration (FDA) of the USA approved FMT for the treatment of recurrent and refractory infection caused by *Clostridioides difficile* [21]. The use of FMT for the therapy of ulcerative colitis dates back to 1988, when the first idiopathic colitis patient was reported, underwent FMT therapy and was cured [22]. While the FMT treatment of patients suffering from *Clostridioides difficile* infection resulted in an immediate curative effect, with the successfulness of treatment reaching 87–90% [23,24,25], many randomised controlled studies aimed at evaluating the effectiveness of FMT treatment of ulcerative colitis reported ambiguous results [24,26,27,28,29]. The evidence of the effectiveness of FMT in the treatment of IBD is not compact, and thus, this therapy has not yet been considered completely explained and consistent. The less impressive results of microbial alternative therapies of IBD could be attributed to the effect of additional factors, such as the method of administration of FMT, application of antibiotics before the therapy, multi-donor or single donor FMT, dosage, microbial profile of the donor and the recipient, nutrition of the patient and seriousness of the IBD disease [18]. Additional studies should be conducted to confirm the encouraging results of FMT therapy that were obtained in recent years by randomised clinical studies conducted on patients suffering from mild to moderate forms of ulcerative colitis [24,26,30]. Future research should focus on the studies paying great attention to characterisation of microbial and molecular components of the stool of donors and recipients before and after FMT treatment with the aim of improving the selection of donors in favour of those who have their gut microbiota especially enriched with specific taxons or microbial products that are deficient in the gut of the respective patient with UC [29].

The aim of this study was to evaluate the modulation and regenerative effects of FMT on the clinical and histopathological response and changes in the gut microenvironment in a pseudo germ-free animal model subjected to chemical induction of mild, moderate and serious forms (activity) of ulcerative colitis with the hope of obtaining new data related to safety and effectiveness of FMT that can contribute to its improved and optimised use.

## 2. Materials and Methods

### 2.1. Animals, Housing Conditions in Gnoto-Facilities and Feed

The in vivo procedure involved specific pathogen-free (SPF) female mice (6 weeks old) of genetic line BALB/c from the breeding facility Velaz s.r.o. (Prague, Czech Republic), altogether 72 animals. The mice were transported to the accredited Laboratory of Gnotobiology of the Department of Microbiology and Immunology, UVMP Kosice, Slovakia (SK U 16016) by air in special transport units. Subsequently, they were transferred to a gnotobiotic rearing facility of the type of EHRET THF 3271IE 101/97 (EHRET Labor-und Pharmatechnik GmbH Co.&KG, Emmendingen, Germany) and into two-piece glove isolators of the type CBC (CBC, Ltd., Madison, WI, USA) comprising 7 to 8 mice/per polypropylene container with the following dimensions: length 365 mm, width 207 mm, height 140 mm. The animal models were fed *ad libitum* irradiated complete mixed feed intended for mice in barrier rearing (Ssniff Spezialdiäten GmbH, Soest, Germany) composed of (kg diet) crude protein 22%, crude fibre 3.9%, crude fat 4.5%, ash 6.7%, calcium 0.7%, phosphorus 0.5%, iron 100 mg, zinc 100 mg, manganese 30 mg, selenium 0.10 mg and copper 5 mg (vitamin D3 2200 IU, vitamin A 28,000 IU, vitamin E 100 mg) and had unlimited access to autoclaved water in glass bottles. The bedding was provided by SSniff H1 505-739727 (Charles River Laboratoire, Écully, France) and was also treated by irradiation. Air exchange between the gnoto-room with central heating and the isolator environment ensured an optimum temperature of 20–24 °C for mice. Filtration of incoming and outgoing air by a filtration system based on HEPA filters (CBC, Ltd., Madison, WI, USA) and regular exchange of bedding ensured optimum relative humidity in the isolators (45–65%). The filtration units of isolators secured a minimum of 10–15 exchanges of air per hour at an overpressure of 50–70 kPa and air flow 8–30 m^3^. The noise level declared by the manufacturer in the gnotobiotic facility did not exceed 45 dB. The regular circadian rhythm inside the isolators was secured by internal artificial neon fixtures, as well as natural outer light. All experimental materials, including distilled water, glass and metal materials, were sterilised by autoclaving at 121 °C and pressure 1.3 MPa for 30 min.

**Figure 1:**
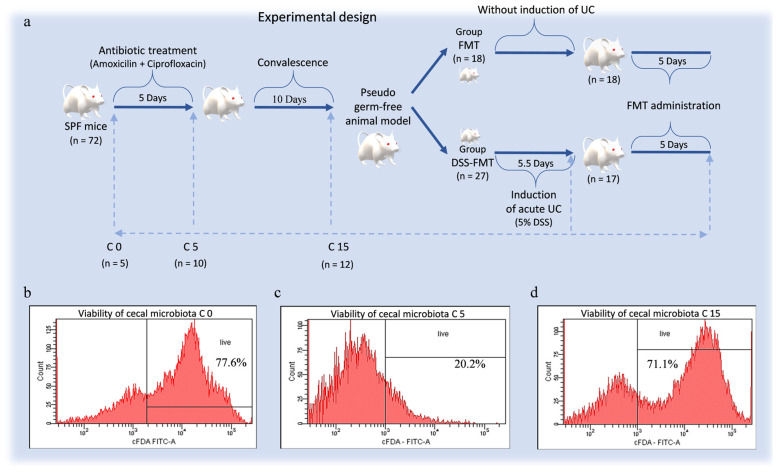
Experimental design and viability of microorganisms in the *caecum* determined by fluorescence-activated cell sorting (FACS). (**a**) Experimental design and timeline. (**b**) Mice *caecum* before the antibiotic treatment (C0, *n* = 6); FACS analysis (77.6%). (**c**) Content of mice *caecum* on day 5 after ATB treatment (C5, *n* = 6); FACS analysis (20.2%). (**d**) Content of mice *caecum* 10 days without ATB (C15, *n* = 6); FACS analysis (71.1%).

In the 1st stage of the procedure, all animals were subjected to a 5-day selective antibiotic decontamination *Amoksiklav* (Sandoz Pharmaceuticals, Ljubljana, Slovenia) and *Ciprinol con infusion* (Krka d.d., Novo Mesto, Slovenia) of their gastrointestinal tract according to the experimental design (Figure 1a). After obtaining the pseudo germ-free (PGF) model with reduced intestinal microbiota, in the 2nd phase of the procedure, the animals were divided into two groups: FMT (*n* = 18 mice) control group without induction of ulcerative colitis and experimental group DSS-FMT (*n* = 27 mice) with induction of acute UC by exposure to 5% DSS (40 kDa, TdB Consultancy AB, Upsala, Sweden) for a period of 5.5 days. As the aim of the study involved observation of microbiological, biochemical, histological and immunological parameters in the digestive tract, at the termination of the procedure, mice were euthanised humanely by means of administration of the preparation Repose 500 mg/mL (*sodium pentobarbital,* Le Vet Beheer, B.V., Oudewater, Nederland) *i.p.* at a dose of 86 mg/kg live weight following the cervical dislocation. Selected mice were euthanised in the period before administration of ATB (C0, *n* = 5); after administration of *Amoksiklav* and *Ciprinol con infusione* (C5, *n* = 10); after 10-day convalescence (C15, *n* = 12); on day 20 of the procedure (after induction of acute UC, *n* = 10); and after FMT treatment (FMT group *n* = 18, DSS-FMT group *n* = 17).

### 2.2. Pseudo Germ-Free Modelling of Animals

The pseudo germ-free animals were obtained by means of administration of broad-spectrum antibiotics to SPF female mice of the BALB/c line using the application scheme (Figure 1a) according to the previously described method by Gancarcikova et al. [31,32]. In the 1st phase of the procedure for obtaining the PGF model, mice with SPF microbiological status were administered perorally *Amoksiklav* 2 × 457 mg/5 mL (Sandoz Pharmaceuticals, Ljubljana, Slovenia) at a dose of 0.2 mL (with active ingredient 387.11 mg/kg/mouse) and subcutaneously the preparation *Ciprinol con infusione* 5 × 10 mL/100 mg (Krka d.d., Novo Mesto, Slovenia) at a dose of 0.1 mL (with active ingredient 19.60 mg/kg/mouse) every 12 h for 5 days while the animals were kept in a strictly defined environment of gnotobiotic isolators. 

Decontamination of the digestive tract of mice with selective antibiotics resulted in the reduction of counts of cultivable microorganisms in the *caecum* content to two morphologically different types of colonies. Investigation of the DNA sequence corresponding to 16S rRNA by BLAST-n analysis showed the greatest coincidence with the species *Escherichia coli* RM9245 (GenBank: CP 044314.1) and *Enterococcus gallinarum* CIFRI-ONUSEG1 (GenBank: MN 481049.1), which corresponded to our previous results [32]. Successful repeatability of the results of identification of bacterial sequences was confirmed at the level of species but with different numbers of the GenBank database. The nucleotide sequences were deposited in GenBank under accession numbers *Enterococcus galinarum* OR939681 and *Escherichia coli* OR939682.

### 2.3. Obtaining the Animal Model with Induced Acute Ulcerative Colitis

Acute ulcerative colitis was induced by chemical exposure to irradiated (Bioster, Veverska Bityska, Czech Republic) synthetic polysaccharide sulphate DSS (dextran sodium sulphate, 40 kDa, TdB Consultancy AB, Upsala, Sweden) according to the previously described method [32], which was added in 5% concentration to autoclaved water supplied to animals for a period of 5.5 days.

### 2.4. Selection of Optimum Human Donor for FMT and Its Processing 

#### 2.4.1. FMT Donor Screening

The safety of the recipients is the main criterion for the selection of the stool donor. The process of selection begins with checking each potential donor, which involves the evaluation of input anamnestic data supplemented with clinical and laboratory examination in order to eliminate the risk of transfer of infectious agents. The collection of samples of biological material from the potential FMT donors conducted within the research project No. 14/2018/OVaV was approved by the Ethical Commission of the University Hospital of L. Pasteur in Kosice, Slovakia. When selecting the optimum donor for human faecal microbiota transplant, the following anamnestic data were considered: Body Mass Index (BMI) that should not exceed 30 and the donor’s lifestyle, including the experience of gastrointestinal disorders such as coeliac disease, irritable bowel syndrome, colitis of different types, inflammatory bowel diseases, infections caused by *Helicobacter pylori*, recent diarrhoea diseases, taking of antibiotics during the past 3 months, use of immunosuppressive medicines, eventual travel to third-world countries within the past 6 months, etc. The complex microbiological (bacterial and virological) and parasitical examination of FMT donors included screening for the blood pathogens *Salmonella*, *Shigella* and *Clostridioides difficile* (GDH and toxins A, B); serological examination for HIV, hepatitis A, B, C and E, *Treponema pallidum*, *Escherichia coli* (enterotoxigenic, enteroinvasive, enteropathogenic, enteroaggregative and strains producing shiga-like toxins), species *Vibrio cholerae* and *Campylobacter* spp.; parasitical examination for *Strongyloides stercoralis*, *Giardia intestinalis*, *Cryptosporidium* spp. and *Entamoeba histolytica*; and virological examination for *cytomegalovirus* (CMV) *rotavirus*, *norovirus* I and II and *adenovirus* 40 and 41. Of the inflammatory markers, the stool of donors was examined immunologically for calprotectin. The human FMT donor used in our study was a 35-year-old male with a BMI index of 24 who fulfilled all the above-mentioned requirements, was a non-smoker and abstinent, did not adhere to a specific dietary pattern, did not visit third-world countries in the past 6 months, and all his selective laboratory examinations provided negative results.

#### 2.4.2. Processing of the Faecal Microbiota Transplant

The stool was processed in a laminary box, safety class II (TelStar, Bio II Advance, Terrassa, Spain), within 1 month at the latest following the screening testing of the donor, and not later than 6 h following the collection of *faeces*. For preparation of the human FMT, we used 50 g of the donated stool, to which we added 250 mL of physiological saline. This mixture was homogenised in a stainless-steel mixer (Waring 7011HS Speed Heavy-Duty Lab Blender, Stamford, CT, USA) in 2 intervals, 4–5 times at 22,000 rev/min. The obtained mixture was filtered through a descending series of laboratory sieves, mesh 2.0 → 1.0 → 0.5 → 0.25 mm, in order to remove non-digested particles of larger size. The volume of stool after filtration was centrifuged at 6000 rev/min for 15 min (Hettich™ ROTINA 420 R, Andreas Hettich GmbH & Co. KG, Tuttlingen, Germany) at −4 °C. The supernatant was removed, and the obtained pellets were resuspended in 125 mL of physiological saline. Sterile pharmaceutical glycerol was added as a cryoprotectant at 10% final concentration in FMT. The processed FMT was stored in 5 mL cryogenic vials (TruCool^®^ Cryogenic Vials, BioCision LLC., Mill Valley, CA, USA) at −70 °C until use.

### 2.5. Evaluation of Clinical Colitis

To determine the clinical activity of colitis, we used specific criteria such as disease activity index (DAI) according to our previous research [32]. Results are presented in Table 1. 

### 2.6. Hematological Analysis

Blood plasma of mice was collected from *sinus orbitalis* by a Pasteur pipette into tubes containing K3-ethylene diamine tetraacetic acid (K3EDTA), which blocks the coagulation cascade. Analysis was carried out employing an autoanalyser BC-2008 VET (Mindray, Shenzhen, China).

### 2.7. Microbiological Analysis

#### 2.7.1. Microbiological Cultivation

Samples of 0.5 g of biological material (*faeces*, *caecum* content) were collected in a sterile manner. They were homogenised (Stomacher Lab Blender 80, Seward Medical Limited, London, UK) and statically cultured in Petri dishes containing nutrient agar of precisely defined composition. The following culture media were used: tryptic soy agar (Biolife, Milan, Italy) with 5% defibrinated ram blood for aerobic incubation (at 37.5 °C for 24 h) and Schaedler agar (HiMedia Laboratories, Mumbai, India) for incubation of bacteria under anaerobic conditions (at 37.5 °C for 48 h). A chemical method was used to create the anaerobic environment, employing anaerobiosis-producing apparatus to obtain an atmosphere free of elementary oxygen (O_2_) (BBL GasPak ™ Plus, BD, Cockeysville, MD, USA) in a hermetically enclosed anaerostat (BBL GasPak™ EZ Container Systems BD Diagnostics, Cockeysville, MD, USA).

#### 2.7.2. Viability of Microorganisms in the *Caecum*

Evaluation of viability (survival) of bacterial cells in the *caecum* of laboratory mice was carried out by the procedure published in a previous study [32] using a flow cytometer BD FACS Canto (Becton Dickinson and Company, Franklin Lakes, NJ, USA) after incubation of samples with carboxyfluorescein diacetate (Sigma-Aldrich, Saint Louis, MO, USA). The viability of bacteria in percent was expressed in a histogram generated using BD FACS DivaTM software v6.1.3 (BD Biosciences, San Jose, CA, USA).

#### 2.7.3. Identification of Cultivable Bacteria 

DNA was isolated from pure bacterial cultures obtained by microbiological incubation of biological specimens. DNA was extracted according to the manufacturer’s instructions for the commercial kit ZymoBIOMICS DNA Kits (Zymo Research, Irvine, CA, USA). The extracted DNA was amplified by PCR reaction in a thermocycler TProfesional Basic (Biometra GmbH, Göttingen, Germany) using a pair of universal primers: 27F (5′-AGAGTTTGATCMTGGCTCAG-3′) and 1492R (5′-CGGYTACCTTGTTACGACTT-3′). The PCR protocol consisted of 5 min of the so-called hot start at 94 °C, 31 cycles for 1 min at 94 °C, 1 min at 55 °C and 3 min at 72 °C, and a final prolongation step for 10 min at 72 °C. The obtained nucleotide sequences of the gene encoding the 16S subunit of ribosomal RNA were compared with the GenBank database by Blast-n analysis.

#### 2.7.4. Detection of Bacterial Microbiota Composition Based on NGS Amplicon Sequencing

Collected biological samples with a volume of 1 g representing pooled samples of SPF mice *faeces*, *faeces* donor FMT, *caecum* FMT group (*n* = 3) and DSS-FMT group (*n* = 4) were stored at −70 °C until DNA extraction. DNA was extracted by a commercial kit ZR Fecal DNA MiniPrep™ (Zymo Research, Irvine, CA, USA) in accordance with the manufacturer’s instructions. Analytical validation of the concentration of the extracted DNA was carried out using the NanoDrop™ 1000 Spectrophotometer (Thermo Fisher Scientific, Wilmington, NC, USA). Extracted DNA was sent for 16S rRNA gene amplicon library preparation and sequencing to Microsynth AG, Switzerland. The V3–V4 region of the 16S rRNA gene was sequenced on the Illumina MiSeq platform in 2 × 250 bp reads. Raw reads were provided and demultiplexed, and biological primers were removed using Cutadapt [33]. The quality of the obtained reads was inspected using fastQC [34], and further bioinformatic processing was carried out in RStudio (R v4.1.1) using the dada2 package v1.20 [35] with default parameters. Forward and reverse reads were trimmed to 225 and 223 bp, respectively, at a maximum expected error rate of 2, allowing no Ns. Further, these were merged using default parameters, and chimeras were removed with the method “consensus”. A total of 734 amplicon sequence variants (ASVs) and 313,291 sequences were obtained from 10 samples, with an average number of sequences per sample of 31,329 ± 4057. ASVs were taxonomically classified using the SILVA reference database version 138 [36]. Bacterial community analyses were performed using the R packages phyloseq v1.36 [37] and vegan v2.6-2 [38], and plots were visualised using the ggplot2 package v3.3.5 [39]. Alpha diversity indices were calculated employing the total, unfiltered community at the ASV level. Raw reads of the 16S rRNA libraries have been deposited in the NCBI Sequence Read Archive (SRA) under accession number PRJNA1016130.

#### 2.7.5. PCR of Multiplex Protocol for Identification of Genes Encoding Factors of Pathogenicity of *Escherichia coli*

The protocol for the PCR reaction was as follows: 95 °C -3 min (94 °C -30 s, 63 °C -90 s, 72 °C -90 s) 30 cycles, 75 °C -5 min. Strain *E. coli* 11501-O:149 (K88+, LTF+, STb+) with concentration of DNA c = 10 ng/µL used as a positive control. The following primers were used: K88: P1FW 5′-GTA TCT GTC CGA GAA TAT CA-3′ a P1Rev 5′-GTT GGT ACA GGT CTT AAT GG-3′ (expected size of the product 478 bp), LTF: P2Fw 5′-GGC GTT ACT ATC CTC TCT AT-3′ a P2Rev 5′-TGG TCT CGG TCA GAT ATG T-3′ (expected size of the product 274 bp), and STb: P3Fw, 5′-TGC CTA TGC ATC TAC ACA AT-3′ a P3Rev, 5′-CTC CAG CAG TAC CAT CTC TA-3′ (expected size of the product 124 bp). DNA of the examined specimens was isolated by means of DNAzol direct (Molecular research centre, Cincinnati, OH, USA). PCR amplificates were visualised by fluorescence staining of nucleic acids using GelRed (Biotium, Fremont, CA, USA) in a 3% agarose gel separation under UV light.

### 2.8. Histological and Immunohistochemical Analysis

Colons (distal part) from each group of mice (*n* = 5–7/group) were isolated, and the inner content was removed with PBS and fixed in 4% paraformaldehyde in PBS (pH 7.2) for 72 h at 8 °C. After rinsing in tap water, colons were dehydrated using a series of ethanol concentrations and embedded in paraffin blocks. Paraffin sections (7 µm thick) of the colons were further processed following standard procedure and used for histological analysis. A part of the histological sections was stained with Harrison’s haematoxylin and eosin (H&E) to determine the intensity of the inflammation and tissue damage. The second series of sections was stained with 0.9% Alcian Blue solution followed by 0.045% Safranin solution, allowing visualisation of Goblet cells and their secretions (seen as a light blue colour). Then, the sections were dehydrated, cleared and mounted in a Histochoice mounting medium (Amresco LLC, Solon, OH, USA). The five non-overlapping sections for each colon/mouse from each group were used to determine the histological activity index (HAI). Next, morphometric analysis was performed on the length of 50 villi and depth of 50 crypts using an Olympus Microscope BX51 and a Digital Analysis Imaging system “Analysis Docu” (Soft Imaging System 3.0, Prague, Czech Republic). The expression of the proteins PCNA and anti-apoptotic Bcl-xL (B-cell lymphoma-extra large) and inflammation markers iNOS (inducible nitric oxide synthase) and COX2 (cyclooxygenase 2) was assessed immunohistochemically. Antigen retrieval was performed by boiling the slides in citrate buffer for 2 min, followed by the procedure described in our previous study [32]. Briefly, the sections were incubated with the following primary antibodies: anti-PCNA (mouse monoclonal) and anti-Bcl-xL (mouse monoclonal; both at the dilution 1:250) overnight at 4 °C. Incubation with anti-iNOS (mouse monoclonal) and anti-COX2 (mouse monoclonal; both at the dilution 1:50) was at 37 °C for 1 h (all antibodies were purchased from Santa Cruz Biotechnology Inc., Dallas, TX, USA). Incubation with the secondary goat anti-mouse IgG antibodies (Dako REAL™ EnVision™/HRP, Rabbit/Mouse (ENV), ready-to-use, Dako, Denmark) was performed for 2 h at room temperature, and diaminobenzidine (DAB) was used as a chromogen (Dako REAL™ DAB+ Chromogen, Dako, Denmark), resulting in the development of the brown colour reaction. To evaluate the intensity of the immunohistochemical reaction in colon tissue quantitatively, approximately six images from the sections of each examined animal (*n* = 6 for each group) were analysed by using a public-domain ImageJ software v1.8.0 (National Institutes of Health, Bethesda, MD, USA). The outlines of all cells that demonstrated an immunopositive signal were marked manually, and the intensity of the brown immunoreactive reaction products was expressed as the relative optical density (ROD) and was calculated using the formula described by Smolen [40] and in our previous research by Gancarcikova et al. [32].

#### Evaluation of Histopathological Finding

Histological analysis and integrity of mucosa were evaluated and classified by an experienced pathologist in a blinded manner, and the histological score (epithelial erosions, crypt loss, infiltrations of inflammatory cells) was assessed based on the parameters shown in Table 2 according to previous research by Gancarcikova et al. [32]. Goblet cell reduction was determined using morphometric analysis, where we measured the area of positively stained mucus formed by the mixed organisation of intercrypt and crypt plume mucus (Table 2).

### 2.9. RNA Extraction, cDNA Synthesis and Real-Time RT-PCR

The inner content of the colons excised from mice was removed using PBS, and the distal parts (2–3 mm long) were dissected, washed, immersed in RNAlater (Sigma-Aldrich, Saint Louis, MO, USA) and stored at −80 °C. Colonic tissue was homogenised in RiboZol ^TM^ RNA Extraction Reagent (VWR), and total RNA was isolated according to the manufacturer’s instructions. The concentration and purity of RNA were determined using an AstraGene spectrophotometer (Harston, Cambridge, UK). RNAs isolated from mice that received DSS and DSS+FMT were further purified after following the protocol described by Viennois et al. [41] that aimed to remove DSS polysaccharides. Briefly, the RNA was isolated using 8 M lithium chloride and then precipitated in 3 M sodium acetate (pH 5.2) (both from Sigma-Aldrich, Saint Louis, MO, USA). Then, 3 µg of total RNA/sample was reverse-transcribed to produce cDNA using RevertAid H Minus M-MuLV Reverse Transcriptase and 100 pmol of oligo dT primers, and the reaction included 20 U of RNase inhibitor and 1 mM dNTPmix (all from ThermoScientific, Burlington, ON, Canada). In the quantitative RT-PCR analysis of the relative abundance of mRNA species, cDNAs served as the templates in the reactions using iTaq SYBR green master mix (BioRad, Hercules, CA, USA) and oligonucleotide pairs for iNOS, COX2, TNF-α, IL-6, IL-1β, TGF-β, IL-10 and two sets of oligonucleotides for mouse housekeeping gene GAPDH. Melting-curve analysis was used to confirm the amplification of the single product. All RT-PCR was performed on a CFX96 thermocycler (BioRad, Hercules, CA, USA). Ct values were normalised to Ct values for both products of the housekeeping gene and expressed as a relative gene expression utilising the 2^−∆∆Ct^ method. Ct values for control samples from colonic tissue from healthy mice without any treatment (*n* = 5) were used as the calibrator. Then, the mean value ± SD of the relative gene expression for each sample was calculated. The list of primers and their sequences are shown in Supplementary Material (Table S1).

The inner content of the colons excised from mice was removed using PBS, and the distal parts (2–3 mm long) were dissected, washed, immersed in RNAlater (Sigma-Aldrich, Saint Louis, MO, USA) and stored at −80 °C. Colonic tissue was homogenised in RiboZol ^TM^ RNA Extraction Reagent (VWR), and total RNA was isolated according to the manufacturer’s instructions. The concentration and purity of RNA were determined using an AstraGene spectrophotometer (Harston, Cambridge, UK). RNAs isolated from mice that received DSS and DSS+FMT were further purified after following the protocol described by Viennois et al. [41] that aimed to remove DSS polysaccharides. Briefly, the RNA was isolated using 8 M lithium chloride and then precipitated in 3 M sodium acetate (pH 5.2) (both from Sigma-Aldrich, Saint Louis, MO, USA). Then, 3 µg of total RNA/sample was reverse-transcribed to produce cDNA using RevertAid H Minus M-MuLV Reverse Transcriptase and 100 pmol of oligo dT primers, and the reaction included 20 U of RNase inhibitor and 1 mM dNTPmix (all from ThermoScientific, Burlington, ON, Canada). In the quantitative RT-PCR analysis of the relative abundance of mRNA species, cDNAs served as the templates in the reactions using iTaq SYBR green master mix (BioRad, Hercules, CA, USA) and oligonucleotide pairs for iNOS, COX2, TNF-α, IL-6, IL-1β, TGF-β, IL-10 and two sets of oligonucleotides for mouse housekeeping gene GAPDH. Melting-curve analysis was used to confirm the amplification of the single product. All RT-PCR was performed on a CFX96 thermocycler (BioRad, Hercules, CA, USA). Ct values were normalised to Ct values for both products of the housekeeping gene and expressed as a relative gene expression utilising the 2^−∆∆Ct^ method. Ct values for control samples from colonic tissue from healthy mice without any treatment (*n* = 5) were used as the calibrator. Then, the mean value ± SD of the relative gene expression for each sample was calculated. The list of primers and their sequences are shown in Supplementary Material (Table S1).

### 2.10. Statistical Analysis

Correlations of two-sided linear dependencies between diverse variables were determined using the Pearson correlation coefficient. The Pearson correlation coefficient is based on a 95% reliability interval, with *p* < 0.05 considered statistically significant. Statistical analysis was performed using the software GraphPad Prism 5.0 for Windows (GraphPad Software, San Diego, CA, USA). The data obtained from molecular, immunohistochemical, clinical, haematological and histological analyses were evaluated by one-way ANOVA, followed by the Tukey post hoc test using multiple comparison. Significant differences between experimental animals were tested by analysis of variance and non-paired Student *t*-test. Results are expressed as means ± SD. Differences between results were considered significant at least at *p* < 0.05. ANOSIM (analysis of similarity) statistical test was used to calculate the difference between the community composition of samples of the FMT and DSS-FMT groups. The Mann–Whitney non-parametric test was used to calculate the significance of alpha diversity indices between the groups FMT and DSS- FMT and to calculate the significance of the relative abundance of selected bacterial taxa between FMT and DSS-FMT using the vegan R package and GraphPad software.

## 3. Results

### 3.1. Next-Generation Sequencing (NGS) Analysis of Microbiological Composition of Donor′s FMT

Analysis of the NGS data using amplicon sequencing (V3–V4 region of 16S rRNA) revealed that the human stool transplant consisted of six different bacterial phyla (Figure S1b) that together accounted for 98.9% of the faecal microbiota of the donor and included phylum Firmicutes (58.94%), Bacteroidetes (20.68%) and Actinobacteriota (18.47%). Phylum Bacteroidetes was represented mainly by the family Bacteroidaceae (Figure S1c; 17.34%) with dominance of the genus *Bacteroides* (Figure S1d; 17.34%), and Actinobacteriota was represented by the family Bifidobacteriaceae and the genus *Bififobacterium* (Figure S1d; 17.13%). Contrary to Bacteroidetes and Actinobacteriota, representatives of the phylum Firmicutes in the *faeces* of the human donor formed a more diverse mixture of genera (Figure S1d) with dominance of genera of the family Lachnospiraceae (Figure S1c; 39.99%), namely genera (Figure S1d) *Fusicatenibacter* (9.99%), *Blautia* (9.54%), *Anaerostipes* (6.40%), *Coprococcus* (3.31%), *Dorea* (3.19%), *Agathobacter* (2.89%) and *Lachnospira* (1.05%). Of the family Ruminococcaceae (Figure S1c; 9.96%), the predominant genera (Figure S1d) were *Faecalibacterium* (7.25%) and *Subdoligranulum* (2.09%). Of the representatives of phylum Firmicutes, non-classified *Lachnospiraceae* (1.17%) and *Intestinibacter* (1.11%) were also present. Other representatives of *Roseburia* genus, non-classified *Ruminococcaceae, Enterococcus* and *Lachnoclostridium* did not exceed 1% in the human FMT. Analysis of the NGS data revealed that the transplant prepared from human stool did not contain pathogenic species, FMT was not a carrier of pathogenic microorganisms that participate in the development of ulcerative colitis and confirmed the listed transplant giver as a suitable FMT donor for this type of study.

### 3.2. Bacterial Composition of Faeces of Conventional SPF Mice

NGS amplicon sequencing of *faeces* of conventional SPF mice revealed dominant 98.16% representation of two phyla (Figure S1a), Firmicutes (54.15%) and Bacteroidetes (44.01%), followed by members of phyla that have not reached 1% proportion in the faecal microbiota: Proteobacteria (0.83%), Campylobacterota (0.40%), Desulfobacterota (0.33%) and Actinobacteriota (0.24%). The phylum Bacteroidetes (Figure S1a) was represented mainly by families (Figure S1c) Muribaculaceae (13.66%), Marinifilaceae (12.02%), Rikenellaceae (8.95%) and Bacteroidaceae (5.94%), and less frequently by Prevotellaceae (3.24%). The predominant genera (Figure S1d) included *Odoribacter* (12.02%), *Alistipes* (8.38%) and *Bacteroides* (5.94%), and representatives of genera *Alloprevotella* (2.88%) were also present, and genera detected in proportions below 1% were represented by *Rikinella* (0.39%), *Prevotellaceae UCG 001* (0.36%) and *Parabacteroides* (0.20%). Before administration of ATB, the phylum Firmicutes was represented in the microbiota of *faeces* of SPF animals by a mixture of families (Figure S1c), with a predominance of members of Lachnospiraceae (29.95%) and in a lesser amount by Enterococcaceae (7.97%), Lactobacillaceae (4.99%), Oscillospiraceae (4.29%) and Ruminococcaceae (1.19%). We observed the presence of a more diverse group of genera (Figure S1d) with a predominance of *Lachnospiraceae NK4A136* (9.83%), *Enterococcus* (7.97%), *Lachnospiraceae UCG 006* (5.36%) and *Lactobacillus* (4.99%). There were also genera of *Marvinbryantia* (2.67%), ASF 356 (2.31%), *Oscillibacter* (1.78%) and *Lachnoclostridium* (1.6%). These data clearly indicate a sufficient richness of intestinal microorganisms in the SPF mouse line BALB/c and do not confirm the presence of pathogenic microorganisms participating in the pathogenesis of ulcerative colitis. 

### 3.3. Antibiotic Treatment of Animals Negatively Affected the Viability of Caecal Microbiota

After antibiotic decontamination (peroral administration of amoxicillin combined with clavulanic acid and *s.c.* administration of ciprofloxacin), we obtained a pseudo germ-free animal model that exhibited reduced viability of the caecal microbiota of mice (Figure 1b–d) determined by fluorescence-activated cell sorting (FACS). Five-day administration of ATB negatively affected the viability of caecal microbiota, as reflected by a significant decrease (*p* < 0.001) in the viability of bacteria (Figure 1c; 18.15 ± 2.09%) in comparison with the period before administration of ATB (Figure 1b; 77.0 ± 1.14%). Although the broad-spectrum antibiotic approach considerably reduced the majority of bacterial species, a portion of microorganisms persisted in the gut, as confirmed by a significantly higher (*p* < 0.001) viability of caecal microbiota at the level of 64.5 ± 6.05% (Figure 1d) after the 10-day interval following the termination of ATB treatment.

### 3.4. Clinical Evaluation of the Effects of FMT on Acute Colitis 

After obtaining the PGF model with reduced intestinal microbiota, the mice were divided into two groups: FMT without induction of ulcerative colitis and the group of animals (DSS-FMT) with induced acute UC by exposure to 5% DSS for 5.5 days. Due to the individual intake of DSS in potable water by animals, on the basis of the developing individual clinical picture of ulcerative colitis (Table 3) that included rectal bleeding and total loss of weight, the DSS-FMT mice were divided into groups with mild (DSS-FMT/Mi), moderate (DSS-FMT/Mo) and severe forms (activity) (DSS-FMT/S) of the disease.

#### 3.4.1. FMT Alleviates Rectal Bleeding of Animals

**Figure 2:**
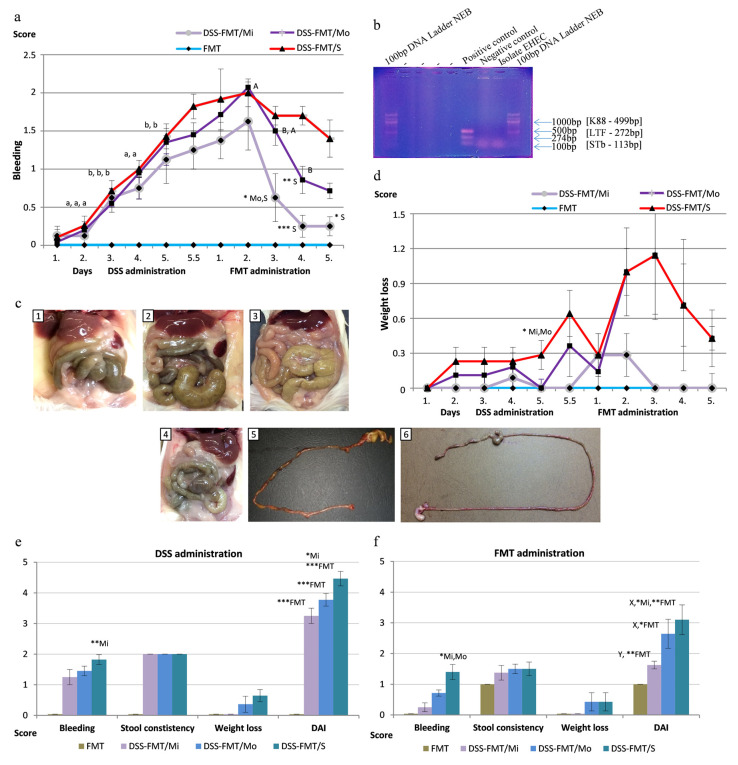
Clinical evaluation of the effect of FMT. (**a**) Rectal bleeding. (**b**) Multiplex PCR negative PCR detection of pathogenicity genes (K88, LT and STba) in translocated *E. coli.* (**c**) (**1**–**6**) Representative figure of post-mortem findings. (**c**) (**1**) Tympanites and *caecum* enlargement at pronounced reduction of gut microbiota following ATB administration. (**c**) (**2**) Megaceacum at dysbiosis on day 10 after ATB administration. (**c**) (**3**) Positive adjustment of dysbiotic microbiota following FMT administration. (**c**) (**4**) Mild UC activity. (**c**) (**5**) Moderate activity of UC enteritis. (**c**) (**6**) Severe activity of UC–enteritis haemorrhagica. (**d**) Weight loss after 5-day exposure to DSS and FMT administration. (**e**) Disease activity index after 5 days following DSS administration. (**f**) Disease activity index after 5 days following FMT administration. FMT (group of animals without UC induction, with administration of FMT, *n* = 18); DSS-FMT/Mi (group of animals with UC induction and FMT treatment–mild form, *n* = 6); DSS-FMT/Mo (group of animals with UC induction and FMT treatment–moderate form, *n* = 9); DSS-FMT/S (group of animals with UC induction and FMT treatment–severe form, *n* = 12). The results are presented as means ± SD. a, b *p* < 0.05; A,B *p* < 0.01 (statistical differences within the group); * *p* < 0.05; ** *p* < 0.01; *** *p* < 0.001 (significant differences between the UC forms). X *p* < 0.05; Y *p* < 0.01; Z *p* < 0.001 (significant differences between DSS periods and FMT administration).

The experimental model of colitis induced by DSS involves rectal bleeding and subclinical haematochezia. Such bleeding is consistent with the mechanism of epithelial damage, particularly if the subepithelial cells were affected by DSS, which results in capillary lesions and loss of blood in the lumen. In the animals with all forms of UC, we observed a significant gradual increase (*p* ˂ 0.05) in the score of rectal bleeding (Figure 2a) from the first to the third day of exposure to DSS. From day 4 to 5.5 of exposure to DSS, the PGF mice with mild UC activity showed a score of rectal bleeding ranging from 0.75 to 1.25 on average, and only slight traces of blood were observed in their *faeces*. According to the clinical picture, only six animals were included in the group with mild disease activity. In mice with moderate (*n* = 9, DSS-FMT/Mo) and severe UC activity (*n* = 12, DSS-FMT/S), rectal bleeding continued to increase significantly (*p* ˂ 0.05) on days 4 and 5 of exposure to DSS, and the score ranged between 0.95 and 1.43. The marked difference between individual forms of UC activity was observed after 5 days and 12 h of exposure to DSS (Figure 2a), with the highest score recorded in mice with serious UC activity (DSS-FMT/S), in which the level of rectal bleeding (1.82) indicated moderate bleeding from the anus. Even after termination of exposure to DSS and the subsequent treatment of animals with human faecal microbiota transplant, rectal bleeding was still detected in the DSS-FMT group, but its intensity was individual (Figure 2a). Up to the second day of FMT administration, all UC forms showed a gradual increase in the score of rectal bleeding, with its highest level of 2.0 reached in DSS-FMT/Mo and DSS-FMT/S, in comparison with mild UC activity (DSS-FMT/Mi) and the score level of 1.6. In the subsequent period, animals from group DSS-FMT/Mi showed a positive effect of treatment with FMT, confirmed by a significantly lower score of rectal bleeding on day 3 of FMT administration (0.6; *p* ˂ 0.05) in comparison with the other two forms of UC and a significantly lower score on day 4 of treatment (0.25; *p* ˂ 0.001) in comparison with the group with severe UC activity. The positive influence of FMT treatment was also observed on days 3 and 4 of its application in mice with moderate UC activity. The rectal bleeding in these animals showed a significant decrease (*p* ˂ 0.01), and its score on day 4 of FMT treatment was significantly lower compared with animals with severe UC activity (0.85; *p* ˂ 0.01). Contrary to this, the score of rectal bleeding in DSS-FMT/S animals remained relatively high until the termination of FMT treatment and ranged between 1.4 and 2. Extensive morphological changes to the intestinal wall are associated with the potential increase in permeability of the intestinal mucosa and the risk of bacterial translocation. Two mice with the severe form of UC died on day 3 of FMT treatment when translocation of *E. coli* was confirmed in the exudate of their abdominal cavity by means of a PCR reaction that amplified the variable region of 16S rRNA (Figure 2b). The obtained sequence of length 1082 bp was subsequently subjected to BLAST-n analysis using the general database GenBank, and 98.24% similarity with *Escherichia coli* registered under Genbank MK621216.1 was detected. The accession number of the DNA sequence deposited in GenBank for *Escherichia coli* was OR939683. On the basis of the PCR test for the presence of pathogenicity genes (K88, LTF, STb) of translocated *E. coli* (Figure 2b), these genes were not confirmed, and the total sepsis was induced by translocation of the non-pathogenic bacterium.

#### 3.4.2. FMT Administration Decreases Total Loss of Weight of Mice Following DSS-Induced Colitis 

The highest weight losses (*p* ˂ 0.05) in animals with serious UC activity were recorded on day 5 of exposure to DSS in comparison with the other two forms of UC, and after the 5.5-day period, the weight loss score reached 0.64, which represented 5–10% total weight loss. The FMT treatment of animals from group DSS-FMT/Mi evoked a similar positive effect toward a decrease in the total weight loss score (Figure 2d) as that observed in the rectal bleeding score. In this group, weight losses at the level of score up to 0.3 were observed only up to day 3 of FMT treatment. An opposite trend was observed in animals from groups DSS-FMT/Mo and DSS-FMT/S, which showed a similar trend of total weight losses that peaked at a score of 1.14 on day 3 of FMT treatment, with weight losses varying around 10%. A more pronounced, although insignificant, adjustment of weight in these groups was detected on day 5 of FMT treatment when the weight loss score decreased to 0.43. 

#### 3.4.3. The Effect of FMT on Adjustment of DAI

The highest disease activity index (DAI, Figure 2e) following the 5.5-day exposure to DSS was observed in animals in the DSS-FMT/S group. These animals exhibited significantly higher DAI (*p* ˂ 0.05) in comparison with those with mild UC activity and reached the 4.6 level on the 10-point scale. In all forms of UC, we recorded a positive effect of FMT treatment on total DAI (Figure 2f), confirmed by a significant decrease in this parameter in moderate and serious UC activity (*p* ˂ 0.05), as well as by a significant decrease in DAI (*p* ˂ 0.01) in DSS-FMT/Mi mice in comparison with the period after exposure to DSS. However, despite this significant decrease, the DAI in DSS-FMT/S animals was relatively high, and because of its high score level (3.25), it was significantly higher (*p* ˂ 0.05) in comparison with the DAI of animals with mild activity and a score of 1.6.

#### 3.4.4. FMT Causes Adjustment in the Pathological–Anatomical Findings of Mice Following DSS-Induced Colitis

One of the important pathological–anatomical findings following the treatment with a faecal microbiota transplant was the adjustment of spleen size observed in 69.8% of animals from the DSS-FMT group in comparison with the period after induction of UC, where splenomegaly was detected at that time in all animals. However, the relative weight of the spleen in DSS-FMT mice was still significantly higher (*p* ˂ 0.05) in comparison with the group without induction of UC (FMT). Similarly, we also observed an adjustment of several-fold enlarged *caecum*, which was attributed to a pronounced reduction of the intestinal microbiota, the size of which was adjusted in 75% of mice (Figure 2c(1–3)). Due to the hepatotoxic and nephrotoxic action of DSS, we observed hypertrophy of the kidneys in 31.2% and of the liver in 18.75% of DSS-FMT animals, also associated with the higher relative weight of the liver (*p* ˂ 0.01) in these animals in comparison with group FMT. The treatment with FMT also resulted in a 21.6% reduction in the occurrence of enteritis (Figure 2c(5,6)), particularly its haemorrhagic form (Figure 2c(6)). 

### 3.5. Effect of FMT on Haematological Parameters of Mice with DSS-Induced Acute Colitis

#### 3.5.1. Haematological Parameters in PGF Animal Model with Induced Acute UC

Systemic inflammatory diseases, such as UC, affect the levels of total circulating white blood cells (WBCs). Investigation of white blood cell counts (Table 4) showed that 5.5-day exposure to DSS resulted in significant differences in all observed parameters between all DSS-FMT forms of UC and the FMT group. 

Haematological analysis revealed total leukocytosis observed particularly in groups with medium (DSS-FMT/Mo) to serious (DSS-FMT/S) forms of ulcerative colitis in comparison with the control FMT group. Significantly high (*p* < 0.05) total counts of WBCs, as well as those of lymphocytes (Lys), were observed in animals with severe UC activity (DSS-FMT/S). Together with a higher proportion of granulocytes (Grans), 2.7-fold higher values in comparison with the group without UC (FMT) indicated an acute inflammatory process in these animals. Increased WBC counts imply active ulcerative colitis and are characteristic of inflammatory lesions infiltrated by them. The ongoing inflammatory process was also confirmed in moderate and mild forms of UC (DSS-FMT/Mo, Mi) but of lower intensity, characterised by significantly (*p* < 0.001) higher total WBC counts compared to FMT that were, however, 2- to 2.6-fold lower than that in the DSS-FMT/S group. In both forms of UC activity (Mi, Mo), significantly (*p* < 0.001) higher Ly counts and higher (*p* < 0.01) Gran and monocyte (Mo) counts were recorded in comparison with the FMT group.

Similar to the above findings, significant differences were observed in red blood cell counts (Table 4) between all UC forms of the DSS-FMT group and the control FMT group. In DSS-FMT/S mice, we confirmed anaemia caused by acute haemorrhage and associated with significantly lower (*p* < 0.001) erythrocyte counts (RBCs), haematocrit level (HCT), concentration of haemoglobin (HGB) and higher mean corpuscular haemoglobin (MCH) compared to FMT group. The loss of blood at colitis flare-up considerably contributes to anaemia and is connected with ulcerative inflammatory changes in the mucosa of the rectum and colon. Activation of thrombocytes in peripheral circulation was increased, and anaemia due to acute haemorrhage was confirmed by pronounced thrombocytosis. Thrombocytes (PLT) as nucleus-free elements with the main role of stopping bleeding and maintaining the integrity of the vascular endothelium were present in DSS-FMT/S in significantly higher (*p* < 0.001) counts, not only in comparison with the FMT group but also compared to animals with mild (DSS-FMT/Mi, *p* < 0.01) and moderate UC activity (DSS-FMT/Mo, *p* < 0.05). An opposite trend was observed in group DSS-FMT/Mo, where in comparison with the FMT group, we recorded significantly increased (*p* < 0.001) counts of RBCs and levels of HCT, HGB and MCH, as well as mean corpuscular volume (MCV), indicating relative polyglobulia due to dehydration. In animals with the mild form of UC (DSS-FMT/Mi), the parameters of red blood cells were at the level determined in the FMT group. In comparison with animals not subjected to UC induction (FMT), the only observed difference was in a significantly higher (*p* < 0.001) proportion of PLT, which indicated an increased load on thrombocytes due to the repair of the damaged vascular endothelium. However, the mild haemorrhage detected in DSS-FMT/Mi mice did not affect the other RBC parameters. This was also confirmed by a significantly lower proportion of PLT (*p* < 0.001, *p* < 0.01) in comparison with UC forms (DSS-FMT/Mo, S).

#### 3.5.2. Positive Effect of FMT on Recovery of Haematological Parameters in Mice with DSS-Induced Acute Colitis

Analysis of white blood cell counts (Table 4) following the 5-day FMT therapy showed significant differences in all investigated parameters between all UC forms of the DSS-FMT group and the FMT group.

In animals with mild and moderate forms of UC, there was a positive effect of FMT treatment in terms of alleviation of the inflammatory response of the animals confirmed by a decrease in WBCs, Lys, Mos and Grans, with significantly reduced counts of WBCs and Lys (*p* < 0.01) in the group with the mild form of UC (DSS-FMT/Mi). An opposite trend was detected in animals with the serious form of UC (DSS-FMT/S), which, despite the reduced count of WBCs in comparison with the period following administration of DSS, showed a significant increase in the proportion of Mos and Grans (*p* < 0.01) in comparison with the FMT group and the proportion of Mos (*p* < 0.05) in comparison with the period following the exposure to DSS that indicated persistence of active inflammation. Despite the positive effect of FMT administration on the recovery of the red blood components in all UC forms, anaemia caused by acute haemorrhage was confirmed in DSS-FMT/S mice resembling that detected after exposure to DSS. This anaemia was associated with significantly lower RBC counts, HGB level (*p* < 0.001), HCT (*p* < 0.05), and significantly increased MCH (*p* < 0.01) in comparison with the FMT group. Although FMT treatment resulted in a positive reduction of PLT in the DSS-FMT/S group, a significantly higher proportion of platelets (*p* < 0.001) compared to the FMT group indicated thrombocytosis and confirmed anaemia caused by acute haemorrhage. An opposite trend was observed in animals with the moderate form of UC. In comparison with the FMT group, they exhibited significantly increased counts of RBCs and levels of HCT, HGB and MCH (*p* < 0.01), as well as those of MCV (*p* < 0.05), which suggested a more promising prognosis of relative polyglobulia or haemoconcentration due to dehydration. However, a significantly lower proportion of thrombocytes (*p* < 0.01), in comparison with the period following the exposure to DSS, suggested ongoing important reparative processes in the intestinal mucosa of animals from the DSS-FMT/Mo group.

### 3.6. Effect of FMT Treatment in the Model of Acute Ulcerative Colitis from the Point of View of Light Microscopy and Histological Activity Index (HAI) of the Disease

#### 3.6.1. Histopathological Features of DSS-Induced Acute Ulcerative Colitis of the Colon

**Figure 3:**
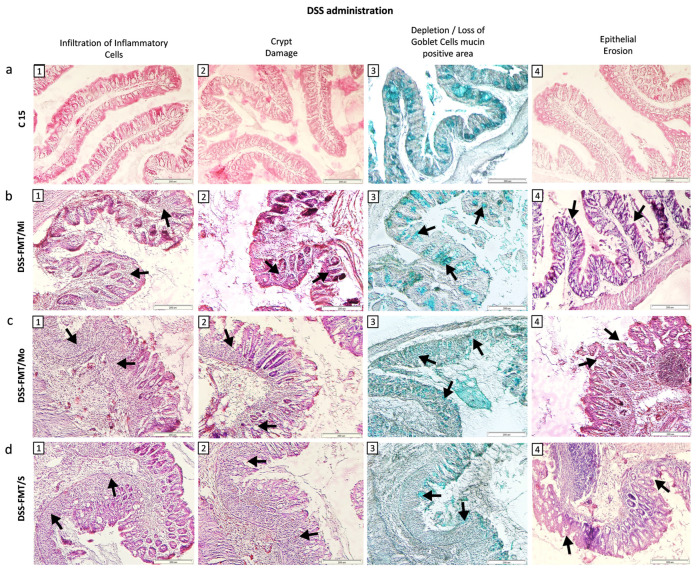
Histological–pathological picture of three clinical forms of chemically induced acute UC activity. (**a**)(**1**–**4**) C15 control group before exposure to dextran sulphate sodium (*n* = 12). (**b**)(**1**–**4**) mild form of UC activity of group DSS-FMT/Mi (*n* = 6). (**c**)(**1**–**4**) moderate form of UC activity of group DSS-FMT/Mo (*n* = 9). (**d**)(**1**–**4**) serious form of UC activity of group DSS-FMT/S (*n* = 12). Infiltration of inflammatory cells, crypt damage, epithelial erosion (arrows; haematoxylin-eosin; ×100); depletion/loss of Goblet cell mucin-positive area (arrows; Alcian blue-safranin + Gillson haematoxylin; ×100).

Comparison of the histological picture of the colon of healthy mice from the C15 group (Figure 3a(1–4)) with histological sections of the colon of experimental group DSS-FMT exposed to 5% DSS revealed considerable damage to the histological structure of *tunica mucosa* and *tela submucosa* (Figure 3b–d). Individual 5.5-day intake of DSS in water by animals resulted in the development of three clinical forms of chemically induced acute ulcerative colitis, confirmed by light microscopy examination of histological sections of mouse colon. The pathophysiological changes in the caudal section of the digestive tract (colon) were defined as a histological activity index (HAI), characterised by evaluation (score) of the presence of epithelial erosion, inflammatory infiltrate, destruction or loss of intestinal crypts and depletion/loss of Goblet cell mucin-positive area. Depletion of mucin, represented by a reduced number and size of Goblet cells, is a significant attribute of acute ulcerative colitis induced by exposure to DSS. Different histopathology of individual clinical forms of induced acute UC observed by light microscopy of histological sections of the colon was also confirmed by qualitative evaluation of their selective morphometric parameters (cross-section area of villi, height and perimeter of villi, depth of crypts and villus height/crypt depth ratio). Mucosa of the colon from mice classified as animals with a mild form of UC (DSS-FMT/Mi) showed a minimum inflammatory response to the chemical agent DSS, characterised by a moderate distribution of neutrophils in the submucosa of colon tissue (Figure 3b(1)). Surface erosions of tunica mucosa associated with damage to intestinal crypts and loss of Goblet cells were present to a minimal extent (Figure 3b(2,3)). Histological pictures of colon sections from the group DSS-FMT/Mo showed active inflammation, confirmed by the presence of the focuses of inflammatory infiltrates localised in *tunica mucosa* and *tela submucosa* (Figure 3c(1,2)). These inflammatory infiltrates were accompanied by serious destructive changes in the surface epithelial layers of the colon. The damage to the integrity of *tunica mucosa* resulted in deformation changes in intestinal crypts (cryptitis, irregular crypts) that were completely absent in certain sections of *tunica mucosa* (Figure 3c(2–4)). Examination of the colon tissue of animals with the serious form of disease (DSS-FMT/S) showed the presence of massive inflammatory focuses that extended beside tunica mucosa and *tela submucosa* and to the *lamina muscularis mucosae* (Figure 3d(1)).

**Figure 4:**
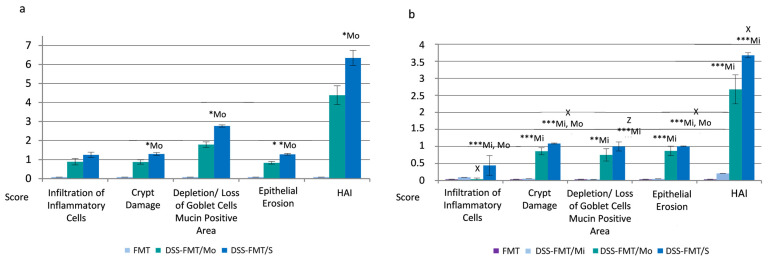
Histological activity index (HAI) of the colon. (**a**) HAI after acute UC induction. (**b**) HAI after 5 days following FMT administration. FMT (group of animals without UC induction, with administration of FMT; *n* = 18); DSS-FMT/Mi (group of animals with UC induction and FMT treatment–mild form; *n* = 6); DSS-FMT/Mo (group of animals with UC induction and FMT treatment–moderate form; *n* = 9); DSS-FMT/S (group of animals with UC induction and FMT treatment–severe form; *n* = 12). The results are presented as means ± SD. * *p* < 0.05; ** *p* < 0.01; *** *p* < 0.001 (significant differences between the UC forms). X *p* < 0.05; Z *p* < 0.001 (significant differences between DSS periods and FMT administration).

The inflammatory response was accompanied by deformation erosions in the colon epithelium associated with loss of intestinal crypts and Goblet cell mucin-positive area (Figure 3d(2–4)). In animals suffering from both forms of UC (DSS-FMT/Mo, DSS-FMT/S, Figure 4a) following the exposure to DSS, there was confirmed a disappearance of Goblet cell mucin-positive area at a score level of 1.78 in animals from group DSS-FMT/Mo, which constituted 25% loss of mucin, and at a score level of 2.76, amounting to 40% loss of mucin in the group DSS-FMT/S. HAI determination in animals with a serious form of the disease indicated a high positive correlation between epithelial erosions and depletion of Goblet cell mucin-positive area (r = 0.8715) and a significant, very high positive correlation between the destruction of intestinal crypts and loss of Goblet cell mucin-positive area (r = 0.9993; *p* < 0.001) that confirmed a significant influence of erosive epithelial damage on depletion of mucin and damage to intestinal crypts.

The toxic effect of DSS on the compactness and integrity of the intestinal mucus barrier in the large intestine was evident in the DSS-FMT/S group, also due to the identification of a high positive correlation between HAI score and bleeding (r = 0.8189). The most pronounced negative impact of the toxic action of 5% polysaccharide of DSS was observed in animals (Table S2) included in the group with the serious form of the disease (DSS-FMT/S) in comparison with the control FMT group with the significantly lowest cross-section of villi and lowest depth of crypts (*p* < 0.001), as well as the lowest height and perimeter of villi (*p* < 0.01). In the DSS-FMT/S mice, we observed a significantly reduced area of the cross-section of villi and of their height (*p* < 0.001) in comparison with both forms of the disease activity (DSS-FMT/Mi, DSS-FMT/Mo), and lower perimeter of villi and depth of crypts (*p* < 0.001) in comparison with mice from the group DSS-FMT/Mi.

The observed pathology of histological architecture of *tunica mucosa* and *tela submucosa* of the colon, manifested by degeneration of the epithelium, loss of mucin, infiltration of *lamina propria* and submucosa with neutrophils and gut cryptitis, accompanied by epithelial erosion, corresponded to the histological picture of DSS-induced acute ulcerative colitis. Histopathology of acute UC was most pronounced in the group with the serious form of the disease (DSS-FMT/S), where a very high positive correlation between DAI and HAI score (r = 0.8189) was detected.

#### 3.6.2. FMT Alters Histological Activity Index of the Disease

**Figure 5:**
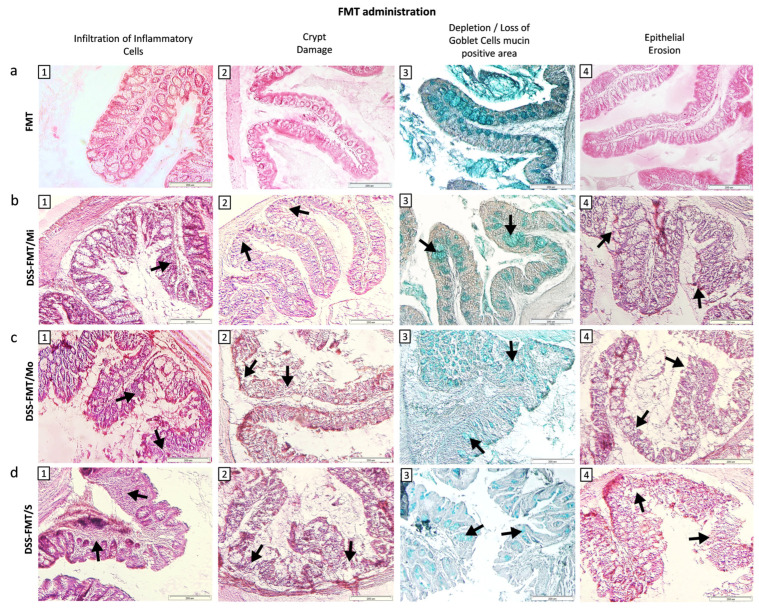
Histopathology of architecture of colon tissue following 5-day treatment with FMT. (**a**)(**1**–**4**) FMT—control group not exposed to DSS and 5-day treatment with FMT (*n* = 18). (**b**)(**1**–**4**) DSS-FMT/Mi—mild form of acute UC activity (*n* = 6). (**c**)(**1**–**4**) DSS-FMT/Mo—moderate form of acute UC activity (*n* = 9). (**d**)(**1–4**) DSS-FMT/S—serious form of acute UC activity (*n* = 12). Infiltration of inflammatory cells, crypt damage, epithelial erosion (arrows; haematoxylin-eosin; ×100); depletion/loss of Goblet cell mucin-positive area (arrows; Alcian blue-safranin + Gillson haematoxylin; ×100).

Light microscopy observation of the colon mucosa of animals classified as the mild form DSS-FMT/Mi and treated with human FMT revealed adjustment of HAI (Figure 5b(1–4)), restoration of the integrity of *tunica mucosa* and absence of epithelial erosions as well as amendment of qualitative morphometric parameters (Table S2). The positive influence of FMT treatment was confirmed by a significantly increased cross-sectional area of villi, height of villi and depth of crypts (*p* < 0.001), not only in comparison with the control group FMT (Figure 5a(1–4)) and both forms of UC (DSS-FMT/Mo, DSS-FMT/S) but also in comparison with the period following induction of acute UC (*p* < 0.05). This positive influence of FMT in animals with a mild form of UC was most likely accentuated by utilisation of the ingested polysaccharide DSS that, at its low doses, could serve as a source of energy for colonocytes. Observation of histological sections from the colon of groups DSS-FMT/S and DSS-FMT/Mo showed that FMT treatment resulted in a reduction in infiltrates of inflammatory cells in both forms. This was confirmed by a significantly lower score in the DSS-FMT/Mo group (Figure 4b; *p* ˂ 0.05) and a positive decrease in HAI score in the group with the serious form of UC (DSS-FMT/S; 0.44) in comparison with the period following the exposure to DSS. The 5-day treatment with FMT positively affected the groups with moderate and serious UC activity also with respect to the recovery of mucin, which was confirmed by a significantly reduced loss of Goblet cell mucin-positive area in the form DSS-FMT/S to the score of 0.99 (Figure 4b; *p* ˂ 0.001), which represented a loss of mucin lower than 20%, and in the form DSS-FMT/Mo, reduction to the score of 0.75. The positive effect of FMT treatment in animals with the DSS-FMT/Mo form was confirmed by the diminution of infiltrates of inflammatory cells (Figure 5c(1)), recovery of mucin and decreased damage to the cylindrical epithelium tunica mucosa by epithelial erosions; however, complete regeneration of the epithelial lining of the colon was not accomplished (Figure 5c(3,4)). Also, complete recovery of selective morphometric parameters was not observed (Figure 5c(2)), which was confirmed by a significantly lower perimeter and the height of villi (*p* < 0.05) and lower cross-sectional area of villi (*p* < 0.01) in comparison with the period of exposure to DSS (Table S2). However, with respect to the cross-sectional area and depth of the crypts, there was some similarity with the control FMT group of mice. In comparison with DSS-FMT/S animals, contrariwise positive, significantly higher values of all investigated parameters (*p* < 0.001) were observed. To a certain extent, a positive effect of FMT treatment towards alleviation of the toxic action of DSS was also observed in animals with the serious form of disease. After FMT treatment of DSS-FMT/S mice, these animals exhibited a reduced score of histological inflammatory infiltrate, and the production of intestinal mucin was also positively affected. However, the score that reflected deformation damage to intestinal crypts was affected only negligibly (Figure 5d(2)), which was confirmed by qualitative evaluation of the remaining morphometric parameters (Table S2) that indicated, in this form, a pronounced damage to all parameters (Figure 5d(1–4)), witnessed by the significantly lower values not only in comparison with the FMT control and both forms of UC but also in comparison with the period after induction of UC (*p* < 0.001).

Histological analysis of the distal section of the digestive tract demonstrated effectiveness of therapy with faecal microbiota transplant in the acute ulcerative colitis model and the contribution of this therapy to the recovery of the observed histological parameters in all three clinical states of UC to the greater or lesser degree. However, our results showed that the treatment with FMT from a healthy donor alone did not result in the expected therapeutic effect in animals with a serious form of UC activity (DSS-FMT/S) in comparison with DSS-FMT/Mi and DSS-FMT/Mo animals.

### 3.7. FMT Modulates Selective Immunohistochemical Markers Associated with Ulcerative Colitis 

Markers of cell proliferation PCNA (proliferating cell nuclear antigen) and cell apoptosis Bcl-xL and inflammatory markers iNOS (nitric oxide synthase) and COX2 (cyclooxygenase) were monitored by immunohistochemical analysis in the tissue of colon with chemically induced ulcerative colitis.

#### 3.7.1. FMT Modulates the Expression of PCNA Marker

**Figure 6:**
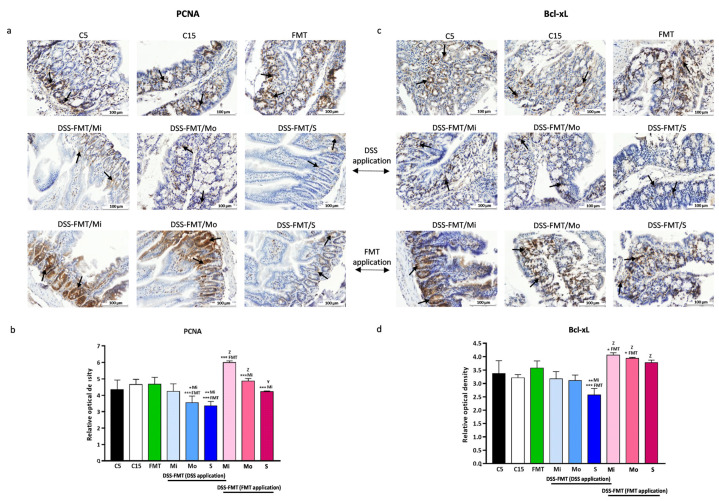
Immunohistochemical detection of markers PCNA and BcL-xL in the intestinal wall of the colon of mice from groups FMT (*n* = 18) and DSS-FMT/Mi/Mo/S (*n* = 6; *n* = 9; *n* = 12). It includes a period of ATB decontamination (C5; *n* = 10), convalescence (C15; *n* = 12) and periods of exposure to DSS and FMT treatment. (**a**) Representative images of intensity of immunohistochemical staining of proliferating cell nuclear antigen (PCNA) at 400× magnification. The arrowhead is positive reaction. (**b**) Quantification of the intensity of immunohistochemical reaction and PCNA expression in mice colon, expressed as relative optical density (ROD) of section. (**c**) Representative images of the intensity of immunohistochemical staining of anti-apoptotic marker (BcL-xL) at 400× magnification. The arrowhead is positive reaction. (**d**) Quantification of the intensity of immunohistochemical reaction and expression of BcL-xL in mouse colon expressed as relative optical density (ROD) of section. The results are presented as means ± SD. * *p* < 0.05; ** *p* < 0.01; *** *p* < 0.001 (significant differences between the UC forms). Y *p* < 0.01; Z *p* < 0.001 (significant differences between DSS periods and FMT administration).

Observation of the immunohistochemical cross-section of mouse colon (Figure 6a) showed that the PCNA-positive cells were located predominantly at the bases of crypts and in *lamina propria*. Quantification of the intensity of the immunohistochemical reaction and PCNA expression in the same section of the digestive tract of mice (Figure 6b), expressed as relative optical density (ROD) of the cross-section, revealed that after exposure to DSS, there was a significantly decreased cell proliferation in the moderate and serious forms of UC, not only in comparison with the FMT group (*p* ˂ 0.001) but also compared to group DSS-FMT/Mi (*p* ˂ 0.01). The negative influence of a 5.5-day exposure to DSS on cell proliferation was also confirmed by the recorded negative medium correlation of the PCNA marker in a serious form of UC (DSS-FMT/S) and histological activity index (HAI, r = −0.690), suggesting a relationship between the increasing histological score and the decreasing cell proliferation. At the same time, a negative medium correlation between the Goblet cell reduction score and the PCNA marker (r = −0.567) was confirmed in this form of UC. The faecal microbiota transplant therapy had a positive effect on the restoration of cell proliferation (Figure 6b), confirmed by comparison with the period of exposure to DSS in all three forms of UC (Mo, Mi *p* ˂ 0.001; S *p* ˂ 0.01). The most pronounced increase in cell proliferation was recorded in the DSS-FMT/Mi form, as in this form, the relative optical density (ROD) of the PCNA marker was significantly higher, not only compared to DSS-FMT/Mo and DSS-FMT/S (*p* ˂ 0.001) forms but also to the group without induction of UC (FMT, *p* ˂ 0.001).

#### 3.7.2. FMT Modulates Expression of Anti-Apoptotic Marker Bcl-xL

One of the important aspects of inflammatory bowel diseases is apoptosis, as it has been associated with their development and progression. It involves a programmable death of the cells that results in the breakdown of excessive and redundant components of cells or the cell as a whole. Protein Bcl-xL is a member of the Bcl-2 family of proteins, within which it plays the role of an anti-apoptotic marker, as it prevents the efflux of cytochrome c from mitochondria and thus hinders apoptosis. In the group DSS-FMT, in all forms of UC induced by DSS, we observed decreased intensity of Bcl-xL expression in the section of mouse colon (Figure 6d), which was significantly decreased, particularly in the form DSS-FMT/S (*p* ˂ 0.001) in comparison with the FMT group. The negative influence of exposure to 5% DSS in a serious form of UC was also confirmed by the high negative correlation between Bcl-xL and HAI score (r = −0.850), as well as by the low negative correlation between the Goblet cell mucin-positive area reduction score and Bcl-xL (r = −0.340). Similar to the observations in marker PCNA, the level of the anti-apoptotic marker Bcl-xL increased following the FMT therapy and its positive stimulation effect was confirmed. The results indicate (Figure 6d) that after transplantation of faecal microbiota in all forms of UC, a significant ROD (*p* ˂ 0.001) of the Bcl-xL marker was observed in comparison with the period of UC induction. Its level was significantly different in DSS-FMT/Mi and DSS-FMT/Mo forms, as well as in comparison with the FMT group (*p* ˂ 0.05).

#### 3.7.3. FMT Modulates Expression of Pro-Inflammatory COX2 Marker

**Figure 7:**
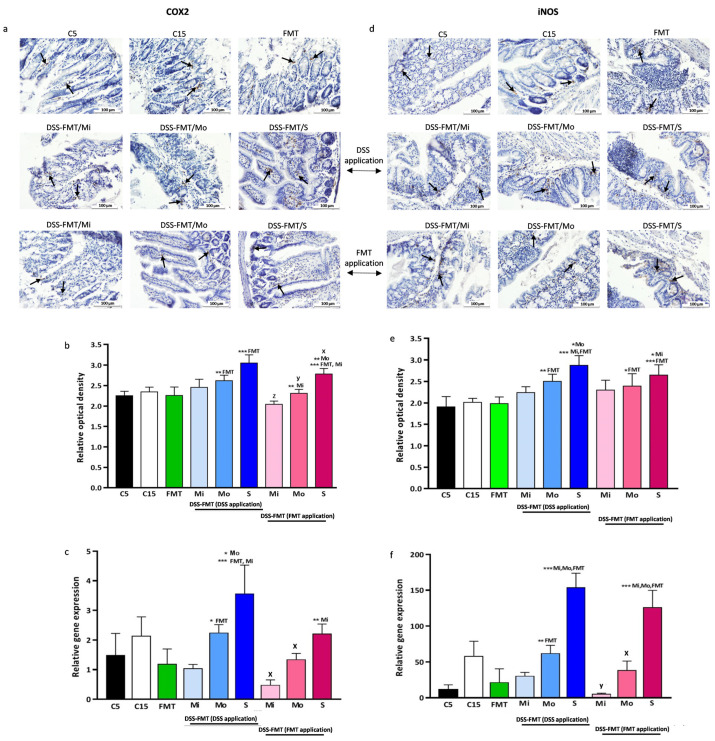
Immunohistochemical detection of markers COX2 and iNOS in the intestinal wall of the colon of mice from groups FMT (*n* = 18) and DSS-FMT/Mi/Mo/S (*n* = 6; *n* = 9; *n* = 12). It includes a period of ATB decontamination (C5; *n* = 10), convalescence (C15; *n* = 12) and periods of exposure to DSS and FMT treatment. (**a**) Representative images of the intensity of immunohistochemical staining of COX2 at 400× magnification. The arrowhead is positive reaction. (**b**) Quantification of the intensity of immunohistochemical reaction and expression of COX2 in mouse colon expressed as relative optical density (ROD) of the section. (**c**) Quantification of the levels of expression of mRNA COX2 by means of reverse transcription-quantitative polymerase chain reaction. (**d**) Representative images of the intensity of immunohistochemical staining of iNOS at 400× magnification. The arrowhead is positive reaction. (**e**) Quantification of the intensity of immunohistochemical reaction and iNOS expression in mouse colon expressed as relative optical density (ROD) of the section. (**f**) Quantification of the levels of expression of mRNA iNOS by means of reverse transcription-quantitative polymerase chain reaction. The results are presented as means ± SD. * *p* < 0.05; ** *p* < 0.01; *** *p* < 0.001 (significant differences between the UC forms). X *p* < 0.05; Y *p* < 0.01; Z *p* < 0.001 (significant differences between DSS periods and FMT administration).

Cyclooxygenase or COX is an enzyme responsible for the production of important mediators called prostanoids, including prostaglandins, prostacyclin and thromboxane. COX1 is considered a constitutive enzyme found in the majority of mammal cells. On the other hand, under physiological conditions, COX2 is not detected in most tissues. It is an inducible enzyme, abundant in activated macrophages and other cells at the site of inflammation. Analysis of the results obtained in our study revealed that induction of acute UC in the DSS-FMT group significantly increased the immunohistochemical response of the COX2 marker (Figure 7a,b) in the colonic tissue in comparison with the FMT group, particularly in the moderate (Mo, *p* ˂ 0.01) and serious forms of UC (S, *p* ˂ 0.001). These results were also supported by increased levels of expression of genes coding the inflammatory protein COX2 (Figure 7c) in DSS-FMT/Mo (*p* ˂ 0.05) and DSS-FMT/S (*p* ˂ 0.001). In the serious form of UC, we recorded significant expression of COX2 marker, also in comparison with the Mi (*p* ˂ 0.001) and Mo forms of UC (*p* ˂ 0.05), and a positive medium correlation between COX2 and a score of epithelial erosion (r = 0.626) was confirmed. After the faecal microbiota transplant, we observed an alleviation of inflammation associated with colitis documented by decreasing levels of this protein in all forms of UC. The most pronounced suppression of expression of COX2 compared to the period of exposure to DSS was detected in the form DSS-FMT/Mi, which exhibited significantly lower levels of optical density of the protein (Figure 7b, *p* ˂ 0.001) as well as its gene expression (Figure 7c, *p* ˂ 0.05). The positive effect of FMT towards reduction of the inflammatory response was also observed in animals with moderate and serious forms of UC. While the immunohistochemical test showed that the intensity of staining of COX2 (Figure 7a,b) was significantly decreased in both forms (Mo, *p* ˂ 0.01; S, *p* ˂ 0.05) in comparison with the period of exposure to DSS, the gene expression of this protein confirmed (Figure 7c) a significant reduction in its level only in the moderate form DSS-FMT/Mo (*p* ˂ 0.05), indicating insufficient suppression of inflammation in the serious form of UC.

#### 3.7.4. FMT Modulates Expression of Pro-Inflammatory iNOS Marker

Endogenous NO develops from L-arginine, namely by oxidation of the terminal nitrogen in a guanidine group in the reaction catalysed by the enzyme nitric oxygen synthase (NOS). iNOS is an inducible isoform of NOS that is highly expressed in macrophages, neutrophils and endothelial cells of smooth muscles at various stimuli. This molecule of protein character is a specific inflammatory marker associated with ulcerative colitis. Animals from the DSS-FMT group with ulcerative colitis induced by dextran sulphate (Figure 7e) exhibited increased levels of inducible nitric oxide synthase (iNOS). A significant increase in the relative optical density of the iNOS marker in comparison with the FMT group was observed, particularly in the moderate (*p* ˂ 0.01) and serious forms of UC (*p* ˂ 0.001). A more pronounced inflammatory response to DSS endotoxin was recorded in the DSS-FMT/S form, with higher levels of the pro-inflammatory marker iNOS (*p* ˂ 0.001, *p* ˂ 0.05) and compared to forms of UC (Mi, Mo). In the altered inflammatory mucosa of animals with induced UC, we observed increased expression of iNOS in DSS-FMT/S (Figure 7f) in comparison with the FMT group (*p* ˂ 0.001) and with both forms of UC (Mi, Mo, *p* ˂ 0.001). The negative influence of the chemical induction of UC in the serious form of colitis was also confirmed by medium positive correlations between the inducible isoform of NOS and the epithelial erosion score (r = 0.643), iNOS and Goblet cell mucin-positive area reduction score (r = 0.627) and iNOS and HAI score (r = 0.561). Following the 5-day faecal microbiota transplant, we observed downregulation of mRNA expression for this protein (Figure 7f), particularly in DSS-FMT/Mi and DSS-FMT/Mo forms (*p* ˂ 0.01, *p* ˂ 0.05), which indicated suppression of inflammation. However, in the DSS-FMT/S form of UC, despite a decrease in the relative gene expression of the pro-inflammatory iNOS marker (Figure 7f), higher mRNA transcript levels were recorded, which were statistically different from the group DSS-FMT/Mi and DSS-FMT/Mo forms of UC (*p* ˂ 0.001), indicating inadequate inhibition of the inflammatory reaction by FMT in the serious form of UC.

### 3.8. FMT Modulated Gene Expression of Cytokines in PGF Animal Model with Induced Acute UC

**Figure 8:**
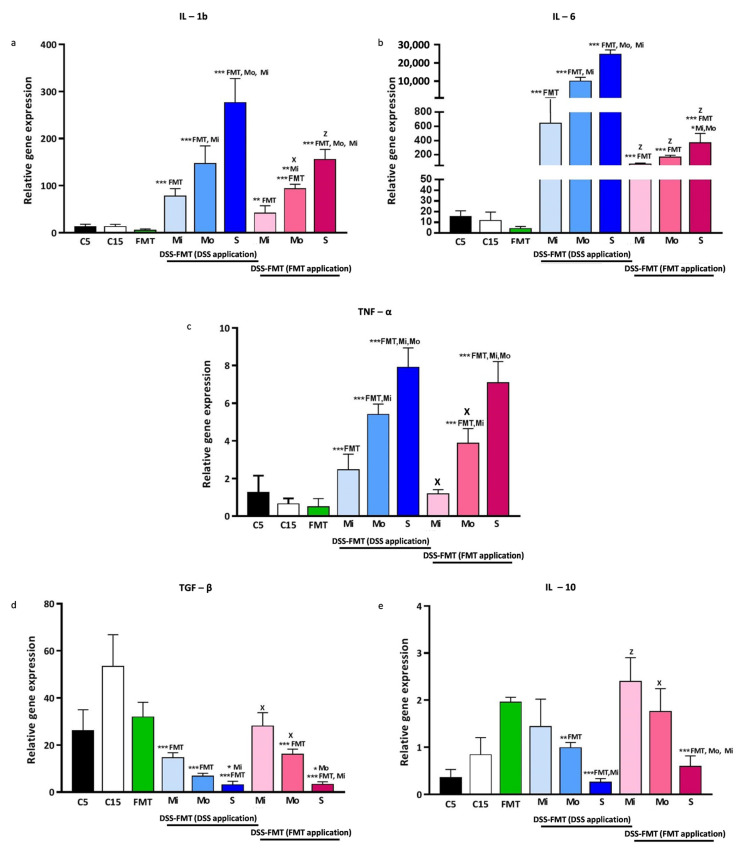
Relative expression of mRNA determined by real-time PCR for (**a**) IL-1β; (**b**) IL-6; (**c**) TNF-α; (**d**) TGF-β; and (**e**) IL-10. C5 (period of ATB decontamination; *n* = 10); C15 (convalescence; *n* = 12); FMT (group of animals without UC induction, with administration of FMT; *n* = 18); DSS-FMT/Mi (group of animals with UC induction and FMT treatment–mild form; *n* = 6); DSS-FMT/Mo (group of animals with UC induction and FMT treatment–moderate form; *n* = 9); DSS-FMT/S (group of animals with UC induction and FMT treatment–severe form; *n* = 12). The results are presented as means ± SD. * *p* < 0.05; ** *p* < 0.01; *** *p* < 0.001 (significant differences between the UC forms). X *p* < 0.05; Z *p* < 0.001 (significant differences between DSS periods and FMT administration).

Cytokines are good indicators of the inflammatory process in the colonic tissue and are produced by various cell types, mainly lymphocytes and macrophages. In the present study, we investigated mRNA transcript levels of pro-inflammatory cytokines IL-1β, IL-6 and TNF-α, as well as regulatory cytokines IL-10 and TGF-β involved in maintaining homeostasis in the colon. We examined gene expression in the colons of mice after antibiotic decontamination (C5), after 10 days of regeneration (C15) in the group subjected to FMT therapy without induction of UC, and in the colons from mice with induction of acute UC by exposure to DSS and subsequent FMT therapy (Figure 8). The 5-day antibiotic decontamination of the digestive tract of mice resulted in a mild increase in IL-1β, Il-6 and TNF-α, which was readjusted after a 10-day regeneration period. The subsequent mild, positive, although insignificant, decrease in these cytokines was also observed in the group subjected to FMT without induction of UC (FMT). However, a 5.5-day exposure to DSS resulted in a significant elevation in gene transcription activity for these cytokines in mild, moderate and severe forms of UC (*p* < 0.001) in comparison with the FMT group. In all forms of UC, we observed the highest transcript levels for IL-6 and IL-1β, and expression of the TNF-α gene was stimulated only moderately. FMT therapy in the group with induction of UC resulted in the attenuation of inflammation in colonic tissue, and transcript levels for these cytokines were lowered, although they were still significantly higher than those found in the FMT group. In the case of IL-1β, FMT treatment (Figure 8a) reduced gene expression in DSS-FMT/Mo (*p* < 0.05) and DSS-FMT/S (*p* < 0.001). IL-6 transcription (Figure 8b) was downregulated after FMT application in all three forms of UC (*p* < 0.001), and TNF-α (Figure 8c) was downregulated in DSS-FMT/Mo (*p* < 0.05) and DSS-FMT/Mi (*p* < 0.05) forms. In the dependence of the DSS intake in water by animals, we observed a significantly (*p* < 0.001) decreased transcription activity of anti-inflammatory cytokine TGF-β (Figure 8d) in comparison with the FMT group, and the lowest mRNA levels were found in the DSS-FMT/S form in comparison with the DSS-FMT/Mi form. However, FMT treatment significantly attenuated the suppressive effect of DSS on TGF- β, particularly in the mild and moderate forms of UC (*p* < 0.05). While the expression of IL-10 (Figure 8e) in colonic tissue was stimulated in the FMT group, the DSS-FMT group showed its decline after induction of acute UC that was the most profound in the DSS-FMT/S form (*p* < 0.001). Similarly, FMT therapy of the DSS-FMT group significantly upregulated mRNA levels in the DSS-FMT/Mi (*p* < 0.001) and DSS-FMT/Mo (*p* < 0.05) forms but not in the DSS-FMT/S form. 

The obtained results suggest that the effect of DSS on cytokine-producing cells is concentration-dependent, and FMT was able to attenuate inflammation below the critical level of tissue damage, in line with the clinical and histological data. 

### 3.9. Effect of FMT on Viability of Caecal Microbiota

**Figure 9:**
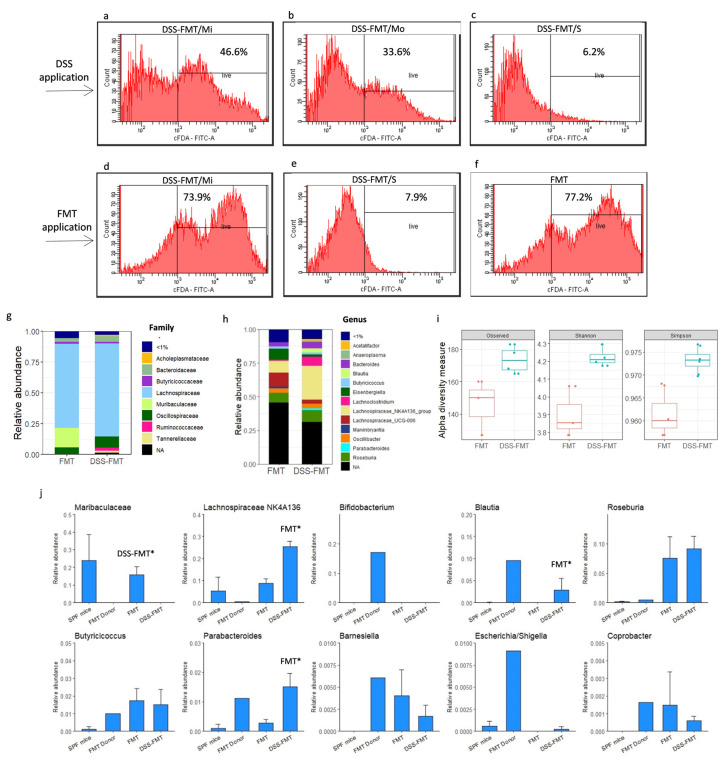
Viability of microorganisms in the *caecum* determined by fluorescence-activated cell sorting (FACS) and NGS analysis of stool samples following administration of FMT (FMT vs. DSS- FMT). Viability after exposure to DSS: (**a**) DSS-FMT/Mi 46.6%, *n* = 3; (**b**) DSS-FMT/Mo 33.6%, *n* = 3; (**c**) DSS-FMT/S 6.2%, *n* = 4. Viability after administration of FMT: (**d**) DSS-FMT/Mi 73.9%, *n* = 3; (**e**) DSS-FMT/S 7.9%, *n* = 6; (**f**) FMT 77.2%, *n* = 6. (**g**) Relative bacterial abundance at family level. (**h**) Relative bacterial abundance at genus level. (**i**) Alpha diversity. (**j**) Relative abundance of selected bacterial taxa on a scale 0–1. Data are shown as average ± SD. Statistical significance was calculated between groups FMT (*n* = 3) and DSS-FMT (*n* = 4) using the Mann-Whitney non-parametric test. Significance shown: * *p* ≤ 0.05.

The picture of DSS-induced acute ulcerative colitis in the DSS-FMT group involving the observed pathology of the histological structure of *tunica mucosa* and *tela submucosa* of the colon was in direct correlation with the decreasing viability of caecal microorganisms, dependent on the degree of activity of UC. While the viability of microorganisms after the convalescent period (Figure 1d) reached a relatively high level (71.1%), the toxic action of DSS in DSS-FMT animals on the integrity of the colonic mucosal barrier was manifested, particularly in the serious form of disease, by complete loss of viability of the caecal microbiota (Figure 9c, 6.2%). The adverse negative action of the chemical agent on the viability of caecal microbiota was also confirmed in the mild and moderate forms of disease (Figure 9a,b), which exhibited significantly lower (*p* < 0.001) levels of the viability of microorganisms (44.6%, 33.6%) in comparison with viability in PGF animals. The FMT treatment of DSS-FMT/Mi animals exhibiting only minimal inflammatory response to a chemical agent (DSS) resulted in the restoration of the integrity of the histological architecture of *tunica mucosa* (Figure 9d), which was also reflected in the significantly higher caecal viability of microorganisms (*p* < 0.001, 73.6%). However, colon tissue in animals from the DSS-FMT/S group showed extensive epithelial erosions, even after the FMT treatment, that were associated with damage to intestinal crypts and subsequent loss of Goblet cells, reflected in the impossibility of colonisation and adherence of microbiota to the intestinal epithelium. The level of viability of caecal microorganisms was still insufficient (Figure 9e, 7.9%). In the animals without induction of UC (Figure 9f), the 5-day FMT treatment favourably affected the viability of their caecal microbiota, which persisted at the level of 77.2%. The results show that the increasing viability of caecal microorganisms was in direct correlation with the restoration of the integrity of the histological architecture of the *tunica mucosa* of the colon. The effectiveness of FMT therapy in induced acute ulcerative colitis on the level of microorganism viability was primarily demonstrated in the mild form of UC. 

### 3.10. Effect of FMT on Composition of Caecal Microbiota in Acute UC Murine Model 

NGS analysis of the composition of the caecal microbiota in the group of DSS-FMT animals with induced ulcerative colitis revealed that their microbiota consisted of the same strains as those detected in group FMT but in different percentage proportions. Comparison of the community composition at the ASV level between the groups FMT and DSS-FMT showed a statistically significant difference and a complete separation of the groups based on Bray–Curtis dissimilarities (ANOSIM: R = 1, *p* = 0.026; Figure S2). While in the *faeces* of conventional SPF mice, a dominant proportion (98.16%) of two strains, Firmicutes (54.15%) and Bacteroidetes (44.01%), was confirmed in the FMT group, the results obtained following the faecal microbiota transplant showed a considerable shift in microbiota in favour of the Firmicutes strain (77.13%) and suppression of the strain Bacteroidetes (18.86%). A still more pronounced difference was recorded in the DSS-FMT group, as the proportion of the strain Firmicutes reached the level of 88.04%, and the proportion of members of the strain Bacteroidetes reached only 6.67%. In the caecal microbiota of FMT animals, the strain Firmicutes was represented by an assortment of families (Figure 9g,h) in average proportions, according to Figure 9h, that point to a dominant (approximately 69%) role of representatives of Lachnospiraceae, less numerous Oscillospiraceae (5.63%) and Butyricicoccaceae (1.73%), and followed by members of families found in lower than 1% proportion in faecal microbiota: Ruminococcaceae and Peptococcaceae. In this group, a higher abundance of genera originating from the initial microbiota of mice was observed (Figure 9i,j), from class Clostridia, with the highest proportion of genera (Figure 9j) non-classified (28.61%), *Lachnospiraceae UCG 006* (10.13%), *Roseburia* (7.57%), *Lachnospiraceae NK4A136* (8.75%) and *Oscillibacter* (3.45%), and genera present in lower than 1% proportion were *Marvynbriantia*, *Lachnoclostridium*, genera *A2* and *ASF 356.* The strain Bacteroidetes was represented (Figure 9g,h) mostly by original families Muribaculaceae (15.66%) and Bacteroidaceae (2.91%) and in a smaller proportion by Marinifilaceae and Rikenellaceae. The prominent genus was non-classified *Muribaculaceae* (15.76%), followed by *Bacteroides* (2.91%), and genera present in proportions lower than 1% were *Odoribacter*, *Parabacteroides* and *Alistipes*. The caecal microbiota of the FMT group was enriched with a diverse mixture of representatives from the FMT donor, which included members of genera *Eisenbergiella* (8.32%), *Butyricicoccus* (1.73%)*, Lachnospiraceae FCS020*, *Akkermansia*, *Anaerostipes*, *Barnesiella*, *Colidextribacter*, *Incertae-Sedis*, *Parasutterella*, *Blautia* and *Coprobacter.* The dysbiosis of PGF mice from the FMT group induced by selective antibiotic decontamination was modified after a 5-day transplantation of the human microbiota; thus, the FMT treatment restored healthy and diverse microbiota. In comparison with the caecal microbiota of the FMT group, the DSS-FMT animals treated with faecal microbiota transplant showed, according to top ten families (Figure 9h), a higher proportion of members of the family Lachnospiraceae (75.21% vs. 68.66%), Oscillospiraceae (8.97% vs. 5.63%), Bacteroidaceae (4.93% vs. 2.91%), Tannerellaceae (1.51% vs. 0.29%), Ruminococcaceae (2.62% vs. 0.77%) and Acholeplasmataceae (1.04% vs. 0.00%). 

While the representatives of the family Muribaculaceae were present in a relatively high proportion (15.66%) in the FMT group, they were absent in the caecal microbiota of animals with induced UC (DSS-FMT, *p* < 0.05). In comparison with the FMT group, within the family Lachnospiraceae (Figure 9j), we particularly detected the members of genera *Lachnospiraceae NK4A136* (*p* < 0.05), *Blautia* (*p* < 0.05), *Roseburia* (9.12% vs. 7.57%) and *Lachnoclostridium* (6.49% vs. 1.01%). 

Members of the genus *Oscillibacter* and non-classified *Lachnospiraceae* were present at the same level in both groups, and the proportion of the latter genus reached circa 23%. Genera *Lachnoclostridium* and *Lachnospiraceae FCS020* showed a moderate increase and, on the contrary, a more pronounced decrease was detected in genera *Lachnospiraceae UCG 006* (2.93% vs. 10.13%) and *Eisenbergiella* (1.71% vs. 8.32%) in comparison with the FMT group. Compared to the FMT group (Figure 9j), in the caecal microbiota of DSS-FMT mice, there was a mild increase in the genus *Bacteroides* (4.93% vs. 2.91%) and participation of genera *Akkermansia*, *Butyricicoccus*, *Acetatifactor*, *Anaerotruncus*, *Adlerocreutzie*, *Bilophile*, *Enterococcus*, *Anaeroplasma*, *Incertae-Sedis*, *Coprobacter*, *Parasutterella*, *Barnesiella* and *Colidextribacter.*

Transplantation of human faecal microbiota in induced acute ulcerative colitis clearly confirmed the restorative effect of FMT application on the composition of intestinal microbiota. The transmission of pathogenic microorganisms was not confirmed in the animals, and there was no multiplication of representatives participating in the development of ulcerative colitis.

## 4. Discussion

The progress in the knowledge of the human intestinal microbiome has shown that there is a relationship between abnormal bowel microbiota and a broad spectrum of disorders and diseases. This knowledge has induced an increasing interest in the scientific community around the world in determining the therapeutic role of FMT [42]. On the basis of current evidence, the transplantation of faecal microbiota is generally considered a safe therapeutic approach with minor adverse effects. The main factors limiting the spread of FMT use in IBD therapy involve minimisation of the risk of infection and transfer of other diseases. The majority of clinical studies or system reviews reported that following FMT, there were some minor temporary undesirable occurrences such as abdominal disorders, diarrhoeas, constipation and mild fever [13]. These side effects are natural responses of the body to the introduction of live microorganisms and their metabolites into the digestive tract. They rapidly disappear and do not present a greater danger to the health of patients [43]. Serious side effects were frequently associated with potential endoscopy complications [15,24]. The side effects of FMT are mainly associated with unsuitable screening of the donor and incorrect analysis of the donor stool material and may result in contamination of the patient with pathogenic microorganisms (viruses, bacteria, parasites, fungi, etc.) and the development of chronic diseases in the recipients [43,44,45] but can even lead to fatal consequences for the recipient in terms of evolvement into total sepsis [21]. The above-mentioned issues indicate that the identification of the optimum donor for FMT is the principal clinical point at issue. Therefore, the selection of the optimum donor for our study was considered the key factor, and the procedure aimed at verification of the beneficial effect of FMT treatment of the PGF animal model with induction of acute UC was carried out using a human donor who satisfied all requirements set by the American Red Cross (see Section 2.4.1. FMT Donor Screening), was a non-smoker and abstinent, did not use a specific diet nor visit any developing country in the past 6 months, and all selective laboratory examinations of this donor provided negative results. At the same time, analysis of the FMT donor by NGS amplicon sequencing confirmed that the FMT was not a carrier of pathogenic microorganisms that participate in the development of ulcerative colitis. Over the past years of the use of FMT treatment, many scientific studies raised additional questions that have not been elucidated as yet but play an important role from the point of view of the successfulness of obtaining exact scientific results. One of them is an insufficient justification of the need for selecting the proper method for the determination of the viability of faecal microbiota. Characteristics of the microbiome in the processed material from the FMT donor are frequently not determined, and if they are, the process usually includes high-efficiency sequencing of the extracted faecal DNA [46]. Such an approach will detect DNA derived from viable and non-viable organisms and, therefore, its ability to indicate which bacteria are viable and able to replicate in the recipient is limited. Cultivation methods can easily isolate only a small subgroup of total intestinal microbiota and, for this reason, are not suitable for the characterisation of the influence of processing of FMT with respect to many present commensal anaerobic species [47]. In our study, we counted the cells by means of flow cytometry with the addition of carboxyfluorescein diacetate, which is decomposed by viable bacteria to fluorescent carboxyfluorescein. However, the method of processing the FMT essentially affects the viability of bacteria. A significant decrease in the proportion of viable bacterial cells was observed after processing in the presence of oxygen and after freezing and defrosting in comparison with anaerobic processing alone. On average, only half of the bacteria were still viable in faecal transplants after immediate processing under strict anaerobic conditions [47], which correlates with our study and the viability of microorganisms in FMT after processing at the level of 44% [48]. This study revealed that homogenisation in a mixer deeply affected the composition of viable bacteria. The increased flow of air associated with high-speed mixing can result in increased exposure to oxygen and can have a harmful effect on species sensitive to oxygen in comparison with manual homogenisation [47]. During our preparation of the material for FMT, we tried to decrease the negative effect of the high rotation speed of the mixer and increased temperature on faecal microbiota by its homogenisation in short, two-second intervals. 

Throughout the past decades, rodents served as model organisms for the analysis of many biological processes and pathological mechanisms. Developed as inbred strains and the product of at least 20 sequential generations of brother–sister mating, these animals show almost 100% homogeneity, i.e., the animals of the same age and sex should be genetically identical, which considerably increases the reproducibility of results and the statistical strength of experiments. Because of its low cost and the relatively easy applicability of the animal model, particularly the DSS-induced model is one of the most frequent models used in the study of various aspects of IBD, such as pathogenesis, genetic predisposition to IBD, immune mechanisms and the role of microbiota in the pathogenesis of IBD [49]. In the presented study, animal murine models with microbiological SPF status of the BALB/c line (females) were used to obtain a PGF model with reduced bowel microbiota and were divided into two groups, namely the control group without induction of ulcerative colitis (FMT) and the DSS-FMT group with induction of acute UC by exposure to 5% DSS for a period of 5.5 days. However, as confirmed by the results of our previous study [32], obtaining the optimum animal model of acute UC by exposure to DSS depends on a number of factors that significantly affect the results, such as the susceptibility of the strain of mice used, molecular weight, concentration of DSS and the length of exposure. Another important factor that has been overlooked in many studies of similar character is the *per os* intake of this concentrated polysaccharide in water that is individual in every animal and results in dependence on DSS uptake and the development of a range of various forms of induced ulcerative colitis. The aim of our study was to evaluate the modulatory and regenerative effects of FMT on clinical and histopathological response and changes in the bowel microenvironment in a PGF animal model with chemical induction of mild, moderate and serious forms (activity) of ulcerative colitis. The individual forms of UC in the animals were identified on the basis of the developing individual clinical picture of ulcerative colitis that included rectal bleeding and loss of total body weight, according to which they were divided into mild (DSS-FMT/Mi), moderate (DSS-FMT/Mo) and serious (DSS-FMT/S) forms (activity) of the disease. The positive effect of FMT treatment in comparison with the period following the exposure to DSS was reflected in a decreased loss of body weight, particularly in the mild form of UC (DSS-FMT/Mi), as in this form, the loss of weight was observed only up to day 2 following the FMT. However, an opposite trend was observed in DSS-FMT/Mo and DSS-FMT/S animals that showed a similar trend of total weight loss, as their weight loss progressed up to day 3 following FMT. In these groups, the increase in weight loss ceased between the third and fourth day following the FMT. Our study also showed that FMT therapy of animals with chemically induced UC positively affected the severity of disease, particularly in DSS-FMT/Mi and DSS-FMT/Mo animals, along with a decrease in the score of total weight loss. There was also observed a significant decrease in DAI and a lowered score of rectal bleeding in DSS-FMT/Mi animals on days 3 and 4 following FMT that corresponded with the adjustment of haematological parameters. Furthermore, we also observed adjustment to the size of the spleen (in 70% of animals), *caecum* (75%), kidneys (31.2%) and liver (18.5%) and a 21.6% decrease in the occurrence of enteritis in all forms of UC, particularly in the haemorrhagic form. Similar results were obtained in the study by [50], as the authors detected significantly higher body weight in mice exposed to DSS and subsequently treated by FMT in comparison with the DSS group without FMT treatment. However, it should be noted that in this study [50], the animals were not divided into individual forms of UC based on their respective clinical picture, and still, despite averaging the body weight of all animals, they were able to detect adjustment of body weight following the FMT treatment. These results corresponded to the conclusions of authors who, in their studies, confirmed that the FMT treatment of model mice of the BALB/c line with induced UC resulted in a positive reduction of total weight losses associated with a significantly lower clinical score of the disease (DAI) and a reduced score of rectal bleeding [9,51,52]. 

Histological changes in the distal section of the colon related to DSS-induced colitis mainly involve the pathology of the histological architecture of *tunica mucosa* and *tela submucosa*, manifested by epithelial degeneration, loss of Goblet cells, infiltration of neutrophils in *lamina propria* and submucosa and intestinal cryptitis, accompanied by epithelial erosion. In addition, submucosal tissue frequently shows notable signs of oedema and hyperplasia [53]. With respect to immune cells, infiltration of colon tissue may be already observed on the first day following induction of UC by DSS, and it increases with time [54]. The pathology of the histological architecture of *tunica mucosa* and *tela submucosa* of the colon, observed in our study, corresponded to the histological picture of DSS-induced acute ulcerative colitis and was most pronounced in the DSS-FMT/S form of disease, which exhibited very high positive correlation between the DAI and HAI score (r = 0.8189), high negative correlation between the anti-apoptotic marker Bcl-xL and HAI score (r = −0.850), moderate positive correlations between inflammatory marker iNOS and score of epithelial erosion (r = 0.643), Goblet cell mucin-positive area reduction (r = 0.627) and HAI score (r = 0.561) and moderate positive correlation between inflammatory marker COX2 and score of epithelial erosion (r = 0.626). Our study confirmed a positive influence of FMT on the reduction of mucosal inflammation in the forms with mild and moderate activity of the disease. Histological sections of the colon showed a decrease in inflammatory cell infiltrates after FMT, which was reflected by a significantly lower score compared to the period of exposure to DSS. Suppression of inflammation was accompanied by recovery of the integrity of *tunica mucosa* and restoration of cell proliferation as demonstrated by PCNA immunoreactivity and supported by the higher level of anti-apoptotic marker Bcl-xL, significantly higher morphometric values of cross-sections of villi, their heights, and the depth of crypts and concurrent absence of epithelial erosions in DSS-FMT/Mi. Reduced inflammatory response of colon mucosa following the FMT treatment that was observed in animals with mild and moderate forms of UC was also documented by decreased expression of inflammatory markers iNOS and COX2, indicating the ongoing reparative processes in colon mucosa of these forms of UC. Similarly, we recorded normalisation of some immunologic parameters in these two forms of UC that was confirmed by the downregulation of mRNA for pro-inflammatory cytokines IL-1β, IL-6 and TNF-α and stimulation of expression of anti-inflammatory and regulatory cytokines IL-10 and TGF-β participating in the maintenance of homeostasis in the colon, accompanied by adjustment of Tc lymphocytes in spleens and Peyer’s patches and NK and NKT cells in spleens [55]. Similar results confirming the protective effect of FMT therapeutic treatment on the adjustment of DAI and HAI scores, manifested by the reduced expression of TNF-α, IL-1p, IL-10, TLR-4 and MyD88 in bowel tissue, were reported by authors who investigated the damage to colon mucosa in mice exposed to DSS [9,51,52,56,57]. The use of FMT as a promising treatment for ulcerative colitis was confirmed by [58] in a clinical study that demonstrated long-term safety and effectiveness of the transplantation of faecal microbiota in patients with ulcerative colitis, namely by reduction of the severity of diarrhoea, haematochezia, Mayo score (stool frequency, rectal bleeding and overall physician’s rating) and EQ-5D index. However, none of the authors conducting experimental studies on animal models mentioned the risk of FMT treatment to animals suffering from serious haemorrhagic symptoms that were present during induced UC. The epithelial surface of the gastrointestinal tract (GIT) is covered by a mucus layer, forming a barrier between intestinal mucosa and the GI content. An important function of this mucus layer is the mechanism of colonisation resistance that provides protection against bacterial colonisation of the GIT [59]. Barrier dysfunction is defined as the loss of a continuous layer of the intestinal epithelium with interruptions in the interepithelial junctions and epithelial gaps [60]. Impaired barrier function increases exposure between intestinal microbiota and epithelial cells, which results in additional stimulation of local immunity and thus contributes to the development of intestinal inflammation [61]. Colon biopsy specimens from patients with UC showed a reduction in the mucus layer. Epithelial barrier disorders associated with changes in the microbial community were observed in patients and experimental models of chronic and acute bowel diseases such as Crohn’s disease, ulcerative colitis [62,63], coeliac disease [64,65,66], intestinal obstruction [67,68] and gastrointestinal infection [69]. Disturbances of the mucus barrier are associated with the potential increase in permeability of the intestinal mucosa and indicate an increased risk of bacterial translocation [70]. In our study, in two mice with high UC activity that died on day 3 of the FMT treatment, there was confirmed translocation of *E. coli* in the exudate of their abdominal cavity. The PCR testing for the presence of *E. coli* pathogenicity genes (K88, LT, STb) provided negative results, and the total sepsis was caused by the translocation of non-pathogenic bacteria. Some medical histories and cohort studies conducted on IBD patients showed a flare-up of IBD in small populations of patients following FMT treatment [71,72]. The definitive mechanisms of the flare-up of IBD following FMT remain unclear, although Quera et al. [72] suggested that transient bacteraemia can result in altered intestinal permeability, resulting in a flare-up of the disease. Some professionals expressed concern that a higher risk of infection may exist in patients with compromised immune systems following FMT treatment. Our study also confirmed that some positive effects of FMT treatment on the toxic action of DSS were also observed in the serious form of UC. However, while in mice from the DSS-FMT/S group, FMT resulted in a decrease in the score of histological inflammatory infiltrate and a mild adjustment in the production of intestinal mucin and PCNA and Bcl-xL markers, the damage to intestinal crypts and the epithelial erosion score were only negligibly affected. Similarly, despite a relative decrease in the activity of pro-inflammatory markers iNOS and COX2 and the mRNA of pro-inflammatory cytokines IL-1β, IL-6 and TNF-α, the levels of these parameters were still relatively high and indicated an insufficient inhibition of the inflammatory reaction by FMT in the serious form of UC. 

A decrease in the diversity [1] and proportions [73] of commensal bacteria, particularly representatives of the strains Firmicutes and Bacteroidetes, is a sign of dysbiosis. The most pronounced is the decrease in the number of beneficial bacterial species of genera *Bacteroides, Lactobacillus*, *Eubacterium* [1,74], *Blautia* and *Roseburia* [75,76] and a relative increase in the proportions of bacterial species of the family Enterobacteriaceae [1,77]. Some studies indicate that the development of IBD takes place with the participation of pathogenic bacteria, the proportion of which increases in IBD, such as *Clostridioides difficile*, enterotoxigenic *Escherichia coli*, *Salmonella* spp. [78,79] and *Fusobacterium* species [73]. Shang et al. [80] reported that a significant positive correlation existed between *Bacteroides*, *Escherichia-Shigella*, *Helicobacter*, *Mucispirillum*, *Clostridiales vadinBB60* group, *Odoribacter*, *Ruminiclostridium* and *Turicibacter* and the inflammation-associated parameters, while a significant negative correlation was recorded for genera *Lachnospiraceae NK4A136 group* and non-classified *Muribaculaceae.* At the same time, a strong correlation was detected between the proportion of the genus *Muribaculaceae* and propionate [81], which can inhibit the activation of CD8+ T cells [82]. This can explain the concurrent negative correlation between *Muribaculaceae* and inflammatory status [80]. A similar parallel was confirmed by our study in the group with induction of UC, where the absence of the genus *Muribaculaceae* was detected in comparison with its considerable proportion in animals from the FMT group. The knowledge related to the significant participation of members of the family Lachnospiraceae in the caecal microbiota of the DSS-FMT group is also positive, particularly due to the association of many species within the group with butyric acid [83] and most of all, the proportion of members with a negative correlation with inflammation, such as the genus *Lachnospiraceae NK4A136* group [80]. After FMT therapy, our study detected the participation of another butyric acid-producing bacterial group in the caecal microbiota, *Roseburia* spp., for which there was confirmed a direct relationship between the pathogenesis of IBD and colon cancer and a significant decrease in counts of its members [84]. Chen et al. [85] showed that the deficit in the bowel microbiota of patients with diagnosed UC or CD is a characteristic feature of their intestinal microbiota. The mentioned proofs stress the importance of butyrate-producing bowel microbiota for intestinal diseases. Analysis of the *caecum* by fluorescence in situ hybridisation (FISH) after 5-day FMT treatment revealed significantly higher (*p* ˂ 0.001) counts of the investigated bacteria *Lactobacillus* spp./*Enterococcus* spp., detected by probe (Lab 158), and *Bifidobacterium* spp., detected by probe (Bif 164), at the level of 6.0–6.2 log_10_ CFU/g in group FMT in comparison with DSS-FMT animals, which was considered a proof of the role of lactobacilli and bifidobacteria from FMT as species beneficial to intestinal protection [86]. At the same time, alpha diversity (Shannon and Simpson index) of caecal microbiota of DSS-FMT mice proved that after FMT treatment, a higher diversity in caecal microbiota was detected in animals with induction of UC in comparison with the control FMT group without induction of UC, which indicated a positive role of microbiota originating from a faecal microbiota transplant. The better outcome of microbiota from FMT in the group with induction of UC was based on more favourable conditions for re-colonisation of faecal microbiota that was supported not only by reduction of the original microbiota of mice by antibiotic decontamination but also by the subsequent toxic action of DSS on caecal microbiota. However, results obtained in our study do not represent all forms of UC, and only animals suffering from the moderate form of UC were used as representative animals for NGS analysis. 

Monitoring of relevant parameters in our study confirmed the effectiveness of the faecal microbiota transplantation therapy in the model of acute ulcerative colitis and contributed in a higher or lower degree to the adjustment of the observed parameters in all three clinical forms of UC. However, our results indicated that an FMT from a healthy donor alone failed to produce the expected therapeutic effect in animals with a serious form of UC activity. We confirmed not only insufficient inhibition of the inflammatory response to FMT in the serious form of UC but also extremely low (approx. 7%) exploitation of viable caecal microorganisms from FMT in this form. The conclusions drawn from our study using animal models are supported by the experience of other authors [18] and also partially by data presented by the studies that confirmed that FMT is most suitable for the treatment of patients suffering from mild to moderate UC, with potential indication as a pre-biological treatment either before initiation of immunomodulatory treatment or as a complementary treatment. Although there also are interesting case reports and case series that were involved in the investigation of the FMT role in serious ulcerative colitis, because of the heterogeneous design of these studies, short-term investigations and a small number of participants, it is difficult to pass judgment on their importance. The results of our study point to safety risks related to the development of bacteraemia and also the translocation of non-pathogenic representatives of bowel microbiota when using FMT in animals diagnosed with a serious form of UC.

Our study met with some limitations. We used a PGF animal model with reduced microbiota and chemical induction of UC. Currently, it is still unclear whether the dysbiotic microbiota in patients with IBD really plays a causative role in the pathogenesis of disease or, alternatively, represents simply a reflection of the inflammatory and antimicrobial responses induced throughout the disease. For this reason, for the purpose of induction of inflammatory disease and evoking more complex pathological conditions in the intestinal ecosystem, the following studies should supplement induction of UC by exposure to DSS by microbiota from a patient with an active form of UC. However, this patient should be an abstinent non-smoker, free of special eating habits, with ulcerative colitis diagnosed for the first time, and not concurrently treated with antibiotics or corticoids nor subjected to biological therapy. Monitoring of relevant specific criteria in individual forms of ulcerative colitis in our study included pathological anatomical findings, evaluation of DAI and HAI scores, haematological parameters supported by morphometric parameters, marker of cellular proliferation PCNA, anti-apoptotic marker Bcl-xL, inflammatory markers iNOS and COX2, mRNA transcript levels of pro-inflammatory cytokines IL-1β, IL-6 and TNF-α, as well as regulatory cytokines IL-10 and TGF-β, and viability of microorganisms. However, in order to ensure a more precise interpretation of the inflammatory process and identification of individual taxonomic members of caecal microbiota to the level of species associated with ulcerative colitis, the subsequent study should be extended to also include NGS analysis of individual forms of UC. Our study presented information about the initial composition of the microbiota of mice before antibiotic decontamination and the caecal microbiota after FMT therapy. However, results of the caecal viability of microorganisms should be supplemented with information provided by NGS analysis of caecal microbiota in individual forms of UC following the chemical induction by DSS. Bacteriophages have the potential to modulate bacterial communities, and their relevance to intestinal diseases or health in mammals remains unclear. Due to their significant participation in the composition of the intestinal microbiota, another limitation of our study is the absence of analyses devoted to the activity of phages associated with UC. 

## 5. Conclusions

Transplantation of human faecal microbiota confirmed the evident restorative effect of FMT treatment on the composition of bowel microbiota. In the experimental animals, there was not a confirmed transfer of pathogenic microorganisms nor detected propagation of representatives participating in the development of ulcerative colitis. In animals with mild and moderate forms of UC, FMT treatment decreased the seriousness of UC, significantly reduced damage to the colon, resulted in a decrease in the clinical and histological index of the disease and reduced the inflammatory response of colon mucosa. However, our results also revealed that FMT from a healthy donor alone failed to achieve the expected therapeutic effect in animals with the serious form of UC activity (DSS-FMT/S) in comparison with animals with DSS-FMT/Mi and DSS-FMT/Mo forms. This observation raises the feasibility of enrichment of FMT with beneficial additives (ideally of natural origin) supporting the healing of colon mucosa, supplying the needed energy substrate to enterocytes and, at the same time, not inducing negative interactions with microbiota contained in the FMT from a healthy donor.

## Figures and Tables

**Table 1:** Disease activity index (DAI) score.

Score	Weight Loss	Stool Consistency	Bleeding	Maximum Score
**0** **1**	No weight loss 5–10%	Formed Mild soft	No bleeding Few blood-tinged stools	10
**2** **3**	11–15% 16–20%	Very soft Watery stool	Slight bleeding Gross bleeding	
**4**	>20%	–	–	

**Table 2:** Histological activity index (HAI) of the colon.

Grade (Score)	Epithelial Erosion	Crypt Damage	Infiltration of Inflammatory Cells	Depletion/Loss of Goblet Cell Mucin-Positive Area
**0**	Morphologically normal	None	Absence of infiltrate	None
**1**	Focal destruction	Some crypt damage, spaces between crypts	Infiltrate at the subepithelial and *lamina propria*	Minimal (<20%)
**2**	Zonal destruction	Large spaces between crypts	Infiltrate reaches *muscularis mucosae*	Mild (21–35%)
**3**	Diffuse and mucosal ulcerations	Large spaces without crypts, surrounded by normal crypts	Severe and extensive infiltrate reaching submocosa and involving *muscularis propria*	Moderate (36–50%)
**4**	–	No crypts	–	Marked (>50%)

**Table 3:** Forms of clinical picture of ulcerative colitis.

Forms		Score	
	Mild (Mi)	Moderate (Mo)	Severe (S)
**Weight loss** **Bleeding**	0–0.3 0–1.25	0.3–0.5 1.0–1.5	0.5–1.0 1.5–2.0

**Table 4:** The levels of haematological parameters in PGF animal model following exposure to DSS and 5-day FMT treatment.

	Group	FMT (*n* = 18)	DSS-FMT/Mi (*n* = 6)	DSS-FMT/Mo (*n* = 9)	DSS-FMT/S (*n* = 12)	Ref BALB/c
after DSS	**WBC (×10^9^/L)**	3.18 ± 0.15	6.26 ± 0.03, *** ^FMT, Y^	8.28 ± 0.77 *** ^FMT^	9.92 ± 3.12 * ^FMT^	3.59–6.40
after FMT	**WBC (×10^9^/L)**	3.28 ± 0.28	5.75 ± 0.05 ** ^FMT, Y^	6.96 ± 0.76 *** ^FMT^	7.30 ± 0.23 * ^Mi,^ *** ^FMT^	
DSS	**Ly (×10^9^/L)**	2.24 ± 0.01	4.20 ± 0.17 *** ^FMT^	5.30 ± 0.24 * ^Mi,^ *** ^FMT^	6.86 ± 1.91 * ^FMT^	2.29–3.59
FMT	**Ly (×10^9^/L)**	2.03 ± 0.18	3.90 ± 0.05 ** ^FMT^	4.90 ± 1.00	3.85 ± 0.54 *** ^FMT^	
DSS	**Mo (×10^9^/L)**	0.28 ± 0.03	0.63 ± 0.08 ** ^FMT^	0.71 ± 0.14 ** ^FMT, X^	0.26 ± 0.08 * ^Mi, X^	0.06–0.62
FMT	**Mo (×10^9^/L)**	0.30 ± 0.03	0.45 ± 0.15	0.33 ± 0.03 ^X^	0.83 ± 0.17 * ^Mo,^ ** ^FMT, X^	
DSS	**Gran (×10^9^/L)**	0.83 ± 0.08	1.43 ± 0.06 ** ^FMT^	2.42 ± 0.56 ** ^FMT^	2.26 ± 0.96	0.74–1.78
FMT	**Gran (×10^9^/L)**	0.95 ± 0.12	1.40 ± 0.10	1.73 ± 0.28 * ^FMT^	2.95 ± 0.68 ** ^FMT^	
DSS	**RBC (×10^12^/L)**	9.53 ± 0.07	9.56 ± 0.17	10.51 ± 0.16 ** ^Mi,^ *** ^FMT^	7.69 ± 0.89 ** ^Mi,^ *** ^Mo, FMT^	8.16–9.98
FMT	**RBC (×10^12^/L)**	9.41 ± 0.10	9.34 ± 0.10	10.34 ± 0.30 ** ^Mi, FMT^	6.41 ± 0.53 *** ^Mo, Mi, FMT^	
DSS	**HGB (g/dL)**	15.26 ± 0.22	15.28 ± 0.32	17.09 ± 0.23 ** ^Mi,^ *** ^FMT^	11.53 ± 0.43 *** ^Mi, Mo, FMT^	12.4–15.4
FMT	**HGB (g/dL** **)**	15.24 ± 0.21	15.02 ± 0.20	16.66 ± 0.37 ** ^FMT, Mi^	12.01 ± 0.62 ** ^Mi,^ *** ^FMT, Mo^	
DSS	**HCT (%)**	46.35 ± 0.29	47.00 ± 1.02	58.10 ± 2.17 ** ^Mi,^ *** ^FMT^	39.83 ± 1.07 ** ^Mi,^*** ^Mo, FMT^	43.5–55.4
FMT	**HCT (%)**	44.38 ± 1.62	44.73 ± 0.78	55.90 ± 2.41 ** ^FMT, Mi^	36.54 ± 1.75 * ^FMT,^ ** ^Mi,^ *** ^Mo^	
DSS	**PLT (×10^9^/L)**	650.5 ± 51.05	1192.00 ± 70.41 *** ^FMT^	1565 ± 32.89 *** ^FMT, Mi, Y^	2048 ± 323.00 *** ^FMT,^** ^Mi,^ * ^Mo^	600–960
FMT	**PLT (×10^9^/L)**	791.50 ± 18.75	1108 ± 178.40 ** ^FMT^	1210 ± 83.49 *** ^FMT, Y^	1607 ± 232.7 *** ^FMT^	
DSS	**MCV (fL)**	48.82 ± 0.51	48.65 ± 0.28	53.36 ± 0.64 *** ^FMT, Mi^	51.90 ± 1.06 * ^Mi, FMT,^	50.8–55.6
FMT	**MCV (fL)**	50.01 ± 0.64	47.88 ± 0.41	54.32 ± 2.36 * ^FMT, Mi^	53.41 ± 0.21 *** ^Mi, FMT^	
DSS	**MCH (pg)**	15.52 ± 0.24	15.73 ± 0.13	16.35 ± 0.08 ** ^Mi,^ *** ^FMT^	16.00 ± 0.40	13–15.5
FMT	**MCH (pg)**	15.94 ± 0.09	16.20 ± 0.10	16.88 ± 0.31 ** ^FMT^	16.88 ± 0.61 ** ^FMT^	

*WBC* white blood cells, *Ly* lymphocytes, *Mo* monocytes, *Gran* granulocytes, *RBC* red blood cells, *HGB* haemoglobin, *HCT* haematocrit, *PLT* platelets, *MCV* mean corpuscular volume, *MCH* mean corpuscular haemoglobin. The results are presented as means ± SD. * *p* < 0.05, ** *p* < 0.01, *** *p* < 0.001 (significant differences between the UC forms). X *p* < 0.05, Y *p* < 0.01, Z *p* < 0.001 (significant differences between DSS periods and FMT administration).

## Data Availability

All the data are contained in the manuscript.

## Supplementary Materials

The following supporting information can be downloaded at: https://www.mdpi.com/article/10.3390/biomedicines12010043/s1, Table S1: List of oligonucleotides and their sequences used in RT-PCR reactions. Table S2: Colon morphology of the PGF mice following 5.5-day exposure to DSS and 5-day administration of FMT. Figure S1: Comparison of composition of bacterial microbiota of PGF mice and FMT from a healthy donor. Figure S2: Non-Metric Multidimensional Scaling (NMDS) plot with Bray–Curtis dissimilarity.
