# Supplementary material for: Beneficial Effect of Faecal Microbiota Transplantation on Mild, Moderate and Severe Dextran Sodium Sulphate-Induced Ulcerative Colitis in a Pseudo Germ-Free Animal Model
Source: Biomedicines. 2023 Dec 22;12(1):43. doi: 10.3390/biomedicines12010043 (PMC10813722; doi:10.3390/biomedicines12010043)
Supplement: Supplementary file 1 [file biomedicines-12-00043-s001.zip › Figure S1 Comparison of composition of bacterial microbiota of PGF mice and FMT from a healthy donor.pdf]

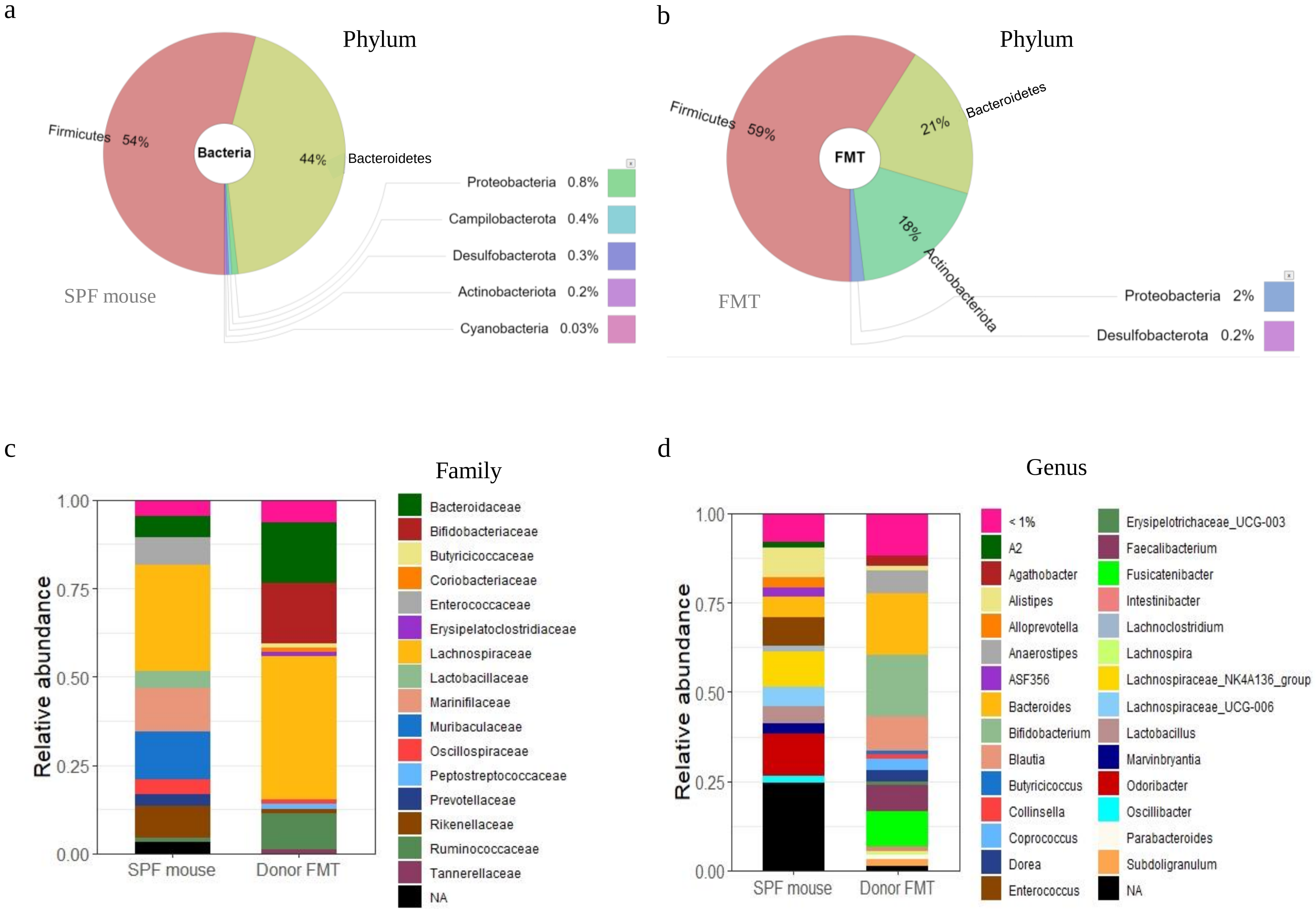

Figure S1. Comparison of composition of bacterial microbiota of PGF mice and FMT from a healthy donor. (a) Krona diagram of PGF mice at the strain level. (b) Krona diagram of FMT at the strain level. (c) Relative abundance of taxons at family level. (d) Relative abundance of taxons at genus level.
