# Supplementary material for: Beneficial Effect of Faecal Microbiota Transplantation on Mild, Moderate and Severe Dextran Sodium Sulphate-Induced Ulcerative Colitis in a Pseudo Germ-Free Animal Model
Source: Biomedicines. 2023 Dec 22;12(1):43. doi: 10.3390/biomedicines12010043 (PMC10813722; doi:10.3390/biomedicines12010043)
Supplement: Supplementary file 1 [file biomedicines-12-00043-s001.zip › Figure S2 Non-Metric Multidimensional Scaling (NMDS) plot with Bray-Curtis dissimilarity.pdf]

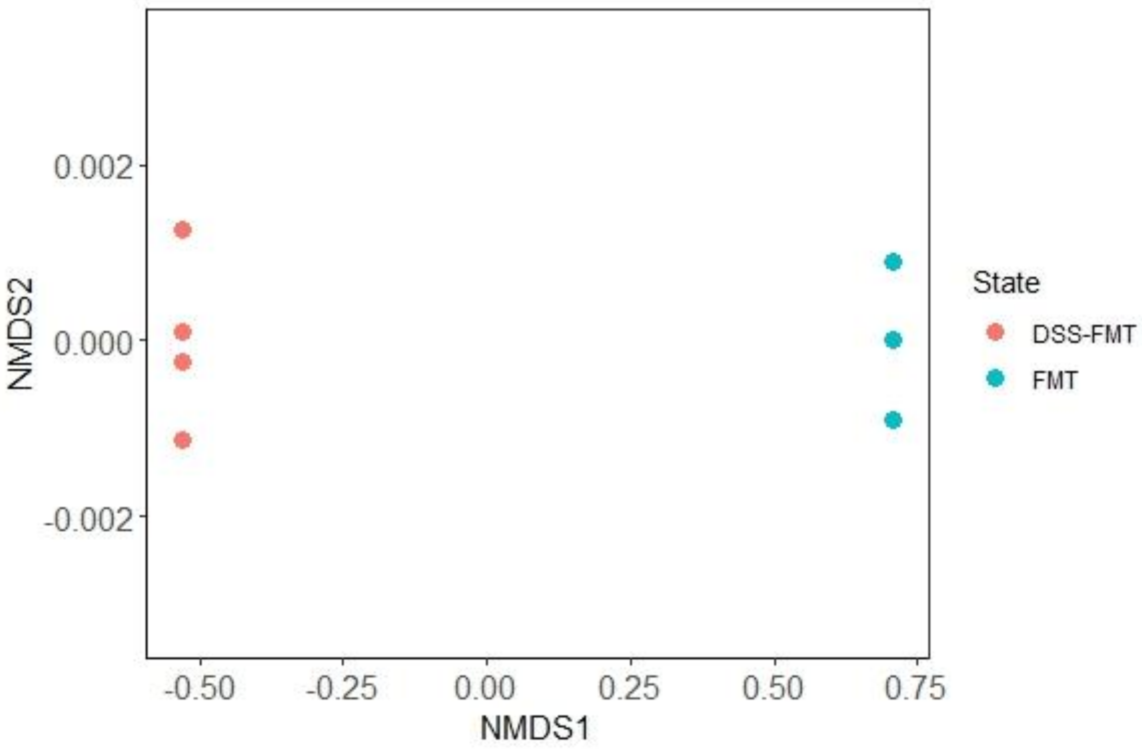

Figure S2: Non-Metric Multidimensional Scaling (NMDS) plot with Bray-Curtis dissimilarity. Plot illustrating relationship of individual samples in groups FMT and DSS-FM. Stress was nearly zero, meaning individual samples within a group share very similar microbiota.
