# Supplementary material for: Beneficial Effect of Faecal Microbiota Transplantation on Mild, Moderate and Severe Dextran Sodium Sulphate-Induced Ulcerative Colitis in a Pseudo Germ-Free Animal Model
Source: Biomedicines. 2023 Dec 22;12(1):43. doi: 10.3390/biomedicines12010043 (PMC10813722; doi:10.3390/biomedicines12010043)
Supplement: Supplementary file 1 [file biomedicines-12-00043-s001.zip › Table S1 List of oligonucleotides and their sequences used in RT-PCR reactions.pdf]

Table S1: List of oligonucleotides and their sequences used in RT-PCR reactions

Gene	Orientation	Sequence
GAPDH	forward	5´-AGGTCGGTGTGAACGGATTTG-3´
	reverse	5´-TGTAGACCATGTAGTTGAGGTCA-3´
iNOS	forward	5′-GCCTCATGCCATTGAATTCATCAACC-3′
	reverse	5′-GAGCTGTGAATTCCAGAGCCTGAA-3′
COX2	forward	5´-TTTGTTGAGTCATTCACCAGACAGAT-3´
	reverse	5´-CAGTATTGAGGAGAACAGATGGGATT-3´
IL-1b	forward	5′ - CTGAATATTTCCCTCCTGGG -3′
	reverse	5′- TCCCGTACAGATGTCCATGAT -3′
TNF-α	forward	5´-GGCAGGTCTACTTTGGAGTCATTGC-3´
	reverse	5´-ACATTCGAGGCTCCAGTGAATTGGG-3´
IL-6	forward	5´-ATGAAGTTCCTCTCTGCAAGAGACT
	reverse	5´-CACTAGGTTTGCCGAGTAGATCTC
TGF-β	forward	5´-TGACGTCACTGGAGTTGTACGG-3´
	reverse	5´-GGTTCATGTCATGGATGGTGC-3´
IL-10	forward	5- CCA AGC CTT ATC GGA AAT GA-3´
	reverse	5´-TTC TCA CCC AGG GAA TTC A-3´
