# Supplementary material for: Beneficial Effect of Faecal Microbiota Transplantation on Mild, Moderate and Severe Dextran Sodium Sulphate-Induced Ulcerative Colitis in a Pseudo Germ-Free Animal Model
Source: Biomedicines. 2023 Dec 22;12(1):43. doi: 10.3390/biomedicines12010043 (PMC10813722; doi:10.3390/biomedicines12010043)
Supplement: Supplementary file 1 [file biomedicines-12-00043-s001.zip › Table S2 Colon morphology of the PGF mice following 5.5-day exposure to DSS and 5-day administration of FMT..pdf]

**Table S2**. Colon morphology of the PGF mice following 5.5-day exposure to DSS and 5-day administration of FMT.

| Group | Cross-Section of Villi μm² | Villus Perimeter μm | Villus Height μm | Crypt Depth μm | Ratio Villus Height/Crypt Depth |
|---|---|---|---|---|---|
| 5.5-day exposure to DSS | | | | | |
| **Colon FMT** | 121 770 ± 4 009 | 1 181 ± 25.10 | 433.8 ± 11.21 | 122.2 ± 2.03 | 3.54 ± 0.12 |
| **DSS-FMT/Mi** | 123 550 ± 4 813 | 1 221 ± 62.55 | 473.3 ± 13.12 | 131.5 ± 7.12 | 3.59 ± 0.25 |
| **DSS-FMT/Mo** | 112 600 ± 5 470 | 1 086 ± 70.00 | 445.8 ± 13.36 | 61.25 ± 0.25 *** FMT , Mi | 7.31 ± 0.05 *** FMT |
| **DSS-FMT/S** | 92 340 ± 2 962 *** FMT, Mo, Mi | 904 ± 55.46 *** Mi , ** FMT | 382.4 ± 9.39 *** Mo, Mi, ** FMT | 57.13 ± 0.44 ** Mo, *** FMT, Mi | 6.58 ± 0.24 *** FMT |
| 5-day administration of FMT | | | | | |
| **Colon FMT** | 90 830 ± 1 279 Z | 1 154 ± 27.45 | 449.8 ± 9.03 | 91.86 ± 3.34 Z | 4.896 ± 0.11 |
| **DSS-FMT/Mi** | 130 200 ± 3 462 *** FMT, X | 1 165 ± 15.77 | 558.8 ± 7.59 *** FMT, X | 173.4 ± 0.83 , *** FMT, X | 3.229 ± 0.12 *** FMT |
| **DSS-FMT/Mo** | 92 690 ± 3 330 *** Mi , Y | 995.1 ± 28.95 *** FMT, Mi, X | 407.10 ± 10.41 *** Mi, X | 91.74 ± 1.98 *** Mi, Z | 4.491 ± 0.09 *** Mi |
| **DSS-FMT/S** | 30 770 ± 2 127 *** Mi, Mo FMT, Z | 499.8 ± 18.60 *** Mi, Mo, FMT, Z | 249.3 ± 10.45 *** Mi, Mo, FMT, Z | 70.63 ± 1.69 *** Mi, Mo, FMT, Z | 3.542 ± 0.12 *** FMT |

FMT (group of animals without UC induction, with administration of FMT; n = 18); DSS-FMT/Mi (group of animals with UC induction and FMT treatment – mild form; n = 6); DSS-FMT/Mo (group of animals with UC induction and FMT treatment – moderate form; n = 9); DSS-FMT/S (group of animals with UC induction and FMT treatment – severe form; n = 12). The results are presented as means ± SD. $^{*}p < 0.05$; $^{**}p < 0.01$; $^{***}p < 0.001$ (significant differences between the UC forms). X < 0.05; Y < 0.01; Z < 0.001 (significant differences between DSS periods and FMT administration).
